# Capture of Neuroepithelial-Like Stem Cells from Pluripotent Stem Cells Provides a Versatile System for In Vitro Production of Human Neurons

**DOI:** 10.1371/journal.pone.0029597

**Published:** 2012-01-17

**Authors:** Anna Falk, Philipp Koch, Jaideep Kesavan, Yasuhiro Takashima, Julia Ladewig, Michael Alexander, Ole Wiskow, Jignesh Tailor, Matthew Trotter, Steven Pollard, Austin Smith, Oliver Brüstle

**Affiliations:** 1 Department of Biochemistry, Wellcome Trust Centre for Stem Cell Research, University of Cambridge, Cambridge, United Kingdom; 2 Institute of Reconstructive Neurobiology, LIFE & BRAIN Center, University of Bonn and Hertie Foundation, Bonn, Germany; 3 Institute of Human Genetics, LIFE & BRAIN Center, University of Bonn, Bonn, Germany; 4 Anne McLaren Laboratory for Regenerative Medicine, University of Cambridge, Cambridge, United Kingdom; University of Southern California, United States of America

## Abstract

Human embryonic stem cells (hESC) and induced pluripotent stem cells (iPSC) provide new prospects for studying human neurodevelopment and modeling neurological disease. In particular, iPSC-derived neural cells permit a direct comparison of disease-relevant molecular pathways in neurons and glia derived from patients and healthy individuals. A prerequisite for such comparative studies are robust protocols that efficiently yield standardized populations of neural cell types. Here we show that long-term self-renewing neuroepithelial-like stem cells (lt-NES cells) derived from 3 hESC and 6 iPSC lines in two independent laboratories exhibit consistent characteristics including i) continuous expandability in the presence of FGF2 and EGF; ii) stable neuronal and glial differentiation competence; iii) characteristic transcription factor profile; iv) hindbrain specification amenable to regional patterning; v) capacity to generate functionally mature human neurons. We further show that lt-NES cells are developmentally distinct from fetal tissue-derived radial glia-like stem cells. We propose that lt-NES cells provide an interesting tool for studying human neurodevelopment and may serve as a standard system to facilitate comparative analyses of hESC and hiPSC-derived neural cells from control and diseased genetic backgrounds.

## Introduction

The advent of cell reprogramming has provided new prospects for disease modeling using patient-derived cells [1], [2]. Induced pluripotent stem cells (iPSCs) generated by expression of transcription factors such as Oct4, Sox2, klf4, c-myc, Nanog and lin28 in skin fibroblasts and other adult cell types have been shown to exhibit a pluripotent phenotype similar to that of embryonic stem (ES) cells, providing a basis for the in vitro generation of various somatic cell types relevant for disease (reviewed e.g. in [3], [4]). However, there is increasing evidence that human ESCs and iPSCs vary in propensity to differentiate into specific cell lineages [5], [6], [7]. In order to identify changes attributable to particular genetic alterations in the context of a complex human genetic background, neurological disease modeling will require neural differentiation procedures that provide well-defined populations for standardized analyses. Several reports have described transitional stages during neural differentiation of human ES cells [8], [9], [10]. Despite the efficacy of some of these protocols with particular cell lines, they are difficult to apply in a standardized manner when it comes to comparing neural progeny from different sources and with different genetic backgrounds [6]. Preferably, neural progenitor cells would be captured in a self-renewing state from where they may be extensively expanded and subsequently directed to generate defined neuronal and glial cell types in an efficient manner. We previously utilized in vitro differentiation of human ESC to generate a population of long-term self-renewing neuroepithelial-like stem cells with stable neurogenic properties (lt-hESNSC hereafter called lt-NES cells [11]).

Here we explore whether the generation of lt-NES cells is applicable to iPSCs and may provide a generic system for recruiting human pluripotent stem cells into a common neural phenotype suitable for scaleable and consistent neuronal differentiation.

## Results

### Generation of long-term self-renewing neuroepithelial-like stem cells (lt-NES cells) from different human pluripotent stem cell sources

**Figure 1:**
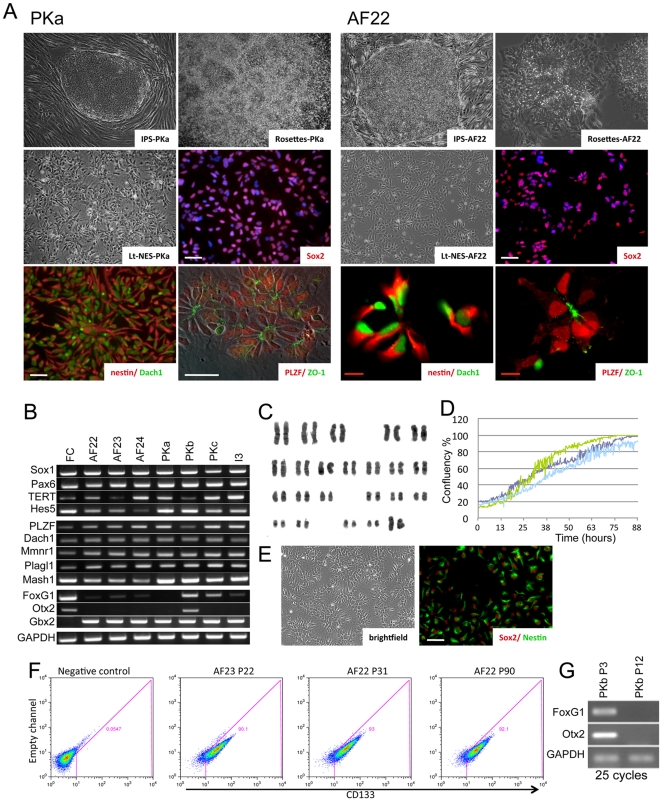
Long-term self-renewing neuroepithelial stem cells (lt-NES cells) derived from pluripotent stem cells of different origins. (**A**) Representative pictures of the lt-NES cell lines PKa and AF22, derived from iPSCs generated from reprogrammed adult dermal fibroblasts. The iPSCs were induced to form neural rosettes, which were isolated and expanded into lt-NES cell lines. Lt-NES cells exhibit a rosette-like growth pattern, stain positive for Sox2, Dach1, Nestin and PLZF and show apical expression of the tight junction protein ZO-1. (**B**) Endpoint RT-PCR analysis (30 cycles) reveals expression of the neural progenitor markers SOX1, PAX6, HES5 and the neural rosette markers PLZF, DACH1, MMNR1 and PLAGL1. Lt-NES cells also express telomerase and high levels of the hindbrain marker GBX2. Fetal human cortex (FC) was used as control. (**C**) Lt-NES cells exhibit a stable karyotype over extensive passaging as assessed by G-banding (line AF22 at passage 30). (**D**) Growth kinetics of the three lt-NES cell lines AF22 (dark blue), AF23 (green), and AF24 (light blue) as measured by change in % confluence over time. (**E**) The clonal lt-NES line AF22:3, derived by single cell deposition into 96-well plates, displays a morphology indistinguishable from its parental line AF22 and stains positive for Sox2 and Nestin. (**F**) The cell surface expression of CD133/PROMININ by AF23 and AF22 lt-NES cells was analyzed using flow cytometry. Debris was excluded by use of TO-PRO-3, and both non-stained cells and cells stained with only the secondary antibody were used to set up the gate (negative control). (**G**) Early passage lt-NES cells (PKb, PKc) still express forebrain markers such as FOXG1 and OTX2. However, expression is lost at higher passages. P, passage number; white scale bars: 100 µm; red scale bars: 10 µm.

To assess the robustness of our approach, studies were performed in parallel in the two laboratories using distinct cell lines. Human iPSC lines were produced by standard retroviral transduction. They are morphologically indistinguishable from human ES cells and express representative markers, including alkaline phosphatase, Tra1-60, Tra1-81, Oct4 or Nanog (Figure 1A and Figure S1).

We applied the lt-NES cell derivation protocol described in Koch et al [11] to three ES cell lines and six different iPSC lines originating from fibroblasts or from radial glia-like neural stem (NS) cells derived from human fetal cortex [12], [13]. We first assessed the capacity of the different pluripotent stem cell lines to give rise spontaneously to neural rosettes following short-term aggregation and subsequent outgrowth culture. All cell lines developed neural rosettes in the outgrowths within 8–12 days (Figure 1A). The frequency of rosette formation varied between the pluripotent cell lines (Table 1), but did not correlate with the donor source (ESC vs. iPSC) or reprogramming method used for iPSC generation. This is of note as parental cells used for reprogramming were both mesenchymal (fibroblasts) and neural, and different transcription factor combinations were used for reprogramming (2–4 factors; Table 1). We thereafter investigated the capacity of the pluripotent cell to give rise to lt-NES cells. Morphologically distinct neural rosettes were manually isolated and cultured in suspension without growth factors for 2–5 days. They were then dissociated into single cells and plated on poly-ornithine and laminin coated plastic in the presence of FGF2 and EGF. From all cell lines and in the majority of trials cells continued to proliferate in adherent culture and over 2–5 passages, acquired a similar appearance and growth pattern. Most remarkably, the cultures exhibited pronounced continuous self-organization into rosette structures as shown in the Movie S1. From passage 5 onwards they were designated lt-NES cell lines. Lt-NES cells could reliably be frozen and recovered using a standard cryopreservation protocol (10% DMSO and 90% culture media). Established lt-NES cell lines were uniformly immunopositive for the neural precursor cell markers Nestin and SOX2 (Figure 1A) and negative for the neuronal and glial differentiation markers, TUJ1 and GFAP, respectively (Figure S2). They expressed transcripts for key neural progenitor cell transcription factors SOX1 and PAX6 (Figure 1B).

**Table 1:** List of lt-NES cell lines, their origins, the reprogramming method used and the capacity of the pluripotent cells lines to form neural rosettes.

Original cell type	lt-NES	Reprogramming factors	Delivery method	Laboratory	Culture medium for PSC propagation	Initial Rosette formation capacity	NES cell passages investigated
ADF	AF22	Oct4, Klf4, Sox2	Retrovirus	Cambridge	DMEM/F12 +20% KSR+10 ng/ml FGF (Preprotech)	Good	102
EDI2 hESC	AF23	N/A	N/A	Cambridge	N2B27 +LIF (1000 U/ml) +50 ng/ml BMP (Preprotech) +10 ng/ml FGF (Preprotech)	OK	80
foetal NSC	AF24	Oct4 and Klf4	Retrovirus	Cambridge	DMEM/F12 +20% KSR+10 ng/ml FGF (Preprotech)	Very good	76
ADF	PKa	Oct4, Klf4, Sox2, c-Myc	VSV-G pseudotyped retroviruses	Bonn	DMEM/F12 +15% KSR+4 ng/ml FGF (Invitrogen)	Very good	46
ADF	PKb	Oct4, Klf4, Sox2, c-Myc	VSV-G pseudotyped retroviruses	Bonn	KnockOut DMEM +20% KSR +50 ng/ml zfFGF (self-made)	Good	35
ADF	PKc	Oct4, Klf4, Sox2, c-Myc	VSV-G pseudotyped retroviruses	Bonn	KnockOut DMEM +20% KSR + 50 ng/ml zfFGF (self-made)	Very good	22
ADF	PKd	Oct4, Klf4, Sox2, c-Myc	VSV-G pseudotyped retroviruses	Bonn	KnockOut DMEM +20% KSR + 50 ng/ml zfFGF (self-made)	Good	43
I3 HESC	I3	N/A	N/A	Bonn	KnockOut DMEM +20% KSR+4 ng/ml FGF (Invitrogen)	Good	121
H9.2 HESC	H9.2	N/A	N/A	Bonn	KnockOut DMEM +20% KSR+4 ng/ml FGF (Invitrogen)	Very good	151

(KSR) knock out serum replacement, (zfFGF2) zebrafish fibroblast growth factor 2, (BMP4) bone morphogenic protein 4, (LIF) leukemia inhibitory factor, (ADF) adult dermal fibroblasts, (hESC) human embryonic stem cells, (NSC) neural stem cells, (VSV-G) vesicular stomatitis virus G protein.

Expansion capacity was investigated by continuous culture of different lt-NES cell lines (Table 1). We did not observe senescence or changes in growth rate for any of the lines for up to 100 passages. Consistent with their extensive proliferative lifespan, lt-NES cell lines showed robust expression of telomerase (Figure 1B). They also expressed HES5 suggesting activity of the Notch pathway in all investigated cell lines [14] (Figure 1B). Metaphase chromosome counts revealed chromosomal stability for more than 1 year of continuous culture (Figure 1C). Live cell imaging was employed to monitor the growth kinetics of lt-NES cells. These studies revealed that all lines investigated have very similar doubling times of 24–30 hours (linear phase, Figure 1D), that remain stable up to at least 62 passages (latest passage examined). The potential to give rise to clonal progeny was investigated by flow cytometric deposition of single cells into individual wells of 96-well-plates coated with human foreskin fibroblasts. Around 10% of the deposited cells from the four tested lines, AF22, AF23, AF24, PKa, developed into proliferating colonies. Clonal lines were expanded from these colonies by passaging in standard lt-NES cell culture conditions without feeders. These clonal lines could be stably propagated for at least 30 passages (latest passage investigated) whilst maintaining a normal chromosome count, consistent rosette-like architecture in culture, and expression of neural progenitor markers Nestin and SOX2 (Figure 1E and data not shown). Thus, growth kinetics, morphology and karyotype are consistent in long-term propagated lt-NES cell lines and clonal isolates.

We next investigated whether lt-NES cells share features of the developing neuroepithelium including polar organization and expression of “rosette markers”, and if these characteristics are conserved across different lt-NES cells generated from ESCs and iPSCs from different genetic backgrounds in different laboratories. We performed immunocytochemical and RT-PCR analyses to investigate the expression of polarity markers and transcription factors typical of neuroepithelial cells [9], [11]. All ESC- and iPSC-derived lt-NES cell lines showed nuclear expression of the transcription factors PLZF and DACH1 (Figure 1A). In addition, consistent expression of MMNR1, PLAGL1 and MASH1 was detected by RT-PCR in 7 lines investigated (Figure 1B). ZO-1, a tight junction marker, which is expressed apically in the neuroepithelium, could be detected at the luminal surface of the rosettes in all lt-NES cell lines, reflecting polarized organization (Figure 1A).

Cell surface markers were investigated by flow cytometry. Lt-NES cells were positive for the neural stem cell marker CD133 (PROMININ [15]; Figure 1F and Figure S3), and analysis of AF22 and AF23 cells showed that this epitope is maintained during long-term culture (up to passage 90). Other surface markers analyzed were PSA-NCAM and CD15 [16] (Figure S3). PSA-NCAM, like CD133 was found on most if not all lt-NES cells. In contrast, only 27% of the cells were positive for CD15. Heterogeneity in CD15 expression has previously been noted in human fetal neural stem cell cultures [12]. The glial progenitor marker A2B5 [17] was detected in less than 10% of lt-NES cells (Figure S3).

Finally, RT-PCR analysis was used to assess potential regional specification of lt-NES cells. All lines investigated (7 out of 9) strongly expressed the posterior marker GBX2. Interestingly, cell lines studied at early passages (PKb; P9, PKc; P10) also showed detectable expression of the anterior transcription factors FOXG1 and/or OTX2 (Figure 1B), but these markers disappeared at later passages (Figure 1G). These findings are in accordance with previous data showing that long-term expanded human ES cell-derived neural stem cells undergo posteriorization with progressive loss of anterior markers from early to later passages [11], [18]. Thus, posteriorization of an initially anterior regional phenotype appears to be a common property for both ESC- and iPSC-derived lt-NES cell lines. It remains to be elucidated whether this process is due to selection or to repatterning induced by continuous exposure to FGF2.

In conclusion, human lt-NES cells from various origins exhibit long-term self-renewal capacity, similar growth kinetics, polar organization, expression of neuroepithelial “rosette markers” and a distinct regional specification with a transcription factor expression profile suggestive of a hindbrain identity.

### Lt-NES cells reliably differentiate into functional neurons and glia

**Figure 2:**
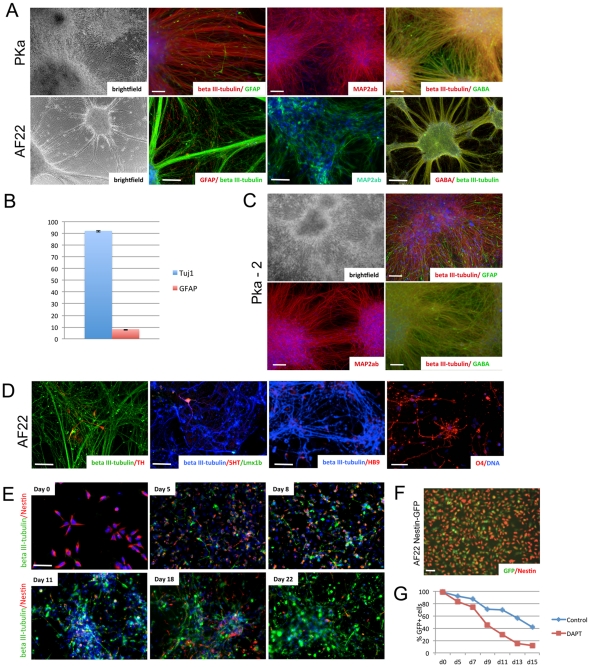
Neuronal differentiation of lt-NES cells. (**A–B**) Four weeks after growth factor removal, lt-NES cells differentiated predominantly into neurons expressing beta III-tubulin (90%), MAP2ab and the neurotransmitter GABA, plus a minor fraction of GFAP-positive astrocytes (10%). Error bars represent STD. (**C**) Following growth factor withdrawal the clonal line PKa-2 gave rise to glia and neurons of preferentially GABAergic phenotype, comparable to the parental line PKa. (**D**) Specific neuronal subtypes expressing TH, 5HT and HB9 could be observed after 4 weeks of differentiation; O4-positive oligodendrocytes were detected after 10 weeks of differentiation. (**E**) AF22 lt-NES cells stained for Nestin and beta III-tubulin at day 0, 5, 8, 11, 18, 22 of differentiation. (**F**) Proliferating AF22 cells expressing GFP under the control of the Nestin enhancer (Nestin-GFP) show double labeling with an antibody to the Nestin protein. (**G**) Nestin-GFP expression in AF22 cells at day 0, 5, 7, 9, 11, 13, 15 of differentiation under control conditions or after exposure to DAPT (2 µM). Scale bars: 100 µm.

To investigate the capacity of the different lt-NES cell lines to give rise to neurons and glia, cells were induced to differentiate by growth factor withdrawal. Over a period of four weeks all lt-NES cell lines differentiated into large numbers of beta III-tubulin-positive neurons (around 90%) and a smaller fraction of GFAP-positive astrocytes (around 10%; Figure 2A–B). Clusters of neurons formed dense networks with extended radially projecting axons. These cells commonly expressed MAP2ab, a marker for more mature neurons (Figure 2A). The neurogenic potential of the lt-NES cells was maintained until at least passage 80 (latest passage investigated for these markers). Most of the neurons expressed the inhibitory neurotransmitter GABA (Figure 2A). This differentiation pattern was also observed in clonal sublines, which similarly gave rise to neurons of predominantly GABAergic phenotype with some glia (Figure 2C). In addition, the cultures contained small numbers of HB9-positive neurons and occasional tyrosine hydroxylase (TH)-positive and serotonergic subtypes (Figure 2D). Glial markers were examined further over a longer time course of differentiation of iPSC- and ESC-derived lt-NES cells (Figure S4). In addition to astrocyte marker expression, O4-positive oligodendrocytes became detectable by ten weeks differentiation (Figure 2D and Figure S4).

Lt-NES cells differentiate asynchronously as shown by the gradual loss of the progenitor marker Nestin and the progressive appearance of the neuronal marker beta III-tubulin (Figure 2E). At the start of differentiation (day 0) over 99% of cells are positive for Nestin, and less than 0.5% express beta III-tubulin. In contrast, by day 22 of differentiation the large majority of cells are beta III-tubulin-positive neurons with only very occasional remaining Nestin-positive cells (Figure 2E). We generated AF22 lt-NES cells stably transfected with a construct carrying green fluorescent protein (GFP) gene under control of the Nestin second intron enhancer [19]. In this line, GFP fluorescence was found to correlate closely to Nestin expression, with virtually all lt-NES cells being GFP positive during proliferation in FGF2 and EGF (Figure 2F–G). Flow cytometry revealed a continuous decrease of Nestin-GFP expression during the first two weeks of differentiation, resulting in only 40% fluorescent cells at day 15 (Figure 2G).

Lt-NES cells expressing the Nestin-GFP reporter might provide a useful system for analyzing the effect of genetic perturbations, growth factors, or small molecules on human neural stem cell self-renewal and differentiation. As a proof-of-concept experiment, we differentiated the Nestin-GFP lt-NES cells in the presence of the gamma-secretase inhibitor *N*-[(3,5-Difluorophenyl)acetyl]-L-alanyl-2-phenyl]glycine-1,1-dimethylethyl ester (DAPT; 2 mM), a known inhibitor of the Notch pathway. Exposure to DAPT resulted in accelerated loss of Nestin-GFP expression with only around 10% of the DAPT-treated cells remaining GFP positive after two weeks of differentiation (Figure 2G). This result is in line with previous data on DAPT exposure of hESC-derived lt-NES cells and provides further evidence that inactivation of Notch signaling accelerates neuronal differentiation [14].

**Figure 3:**
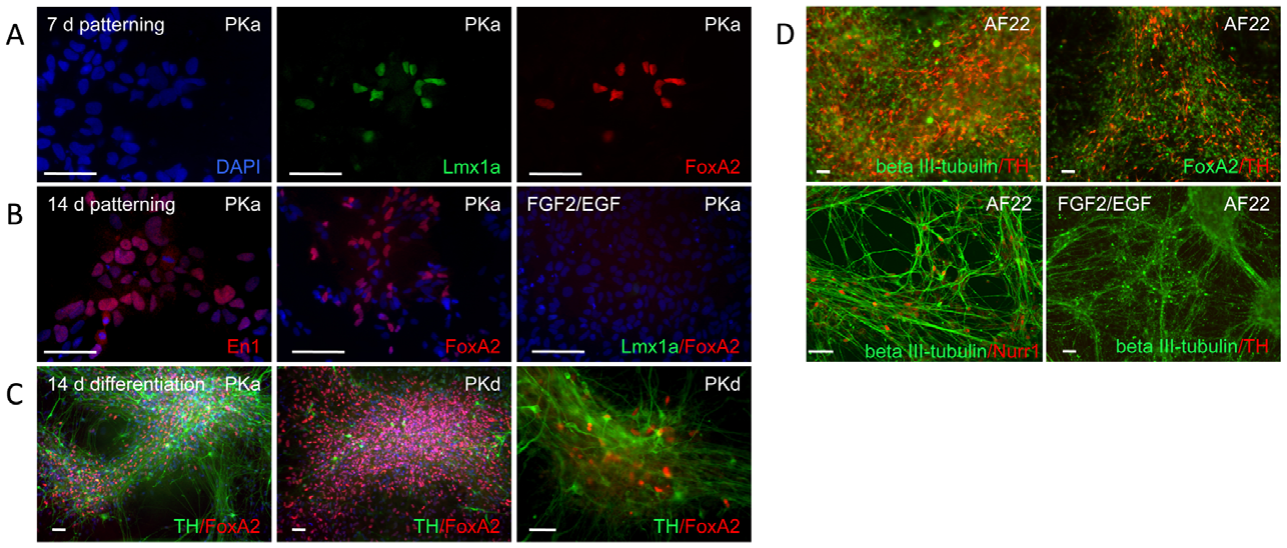
Induction of midbrain dopaminergic neurons using extrinsic factors. IPS cell derived lt-NES cells cultured for 7 days in the presence of sonic hedgehog (Shh) and FGF8 showed nuclear expression of Lmx1a and FoxA2 (**A**). (**B**) With prolonged (14 days) exposure to Shh and FGF8, the number of cells expressing FoxA2 gradually increased. Shh/FGF8-treated cells were also positive for the midbrain marker En1, whereas neither FoxA2 nor Lmx1a were expressed in control populations cultured in the absence of Shh/FGF8. (**C–D**) Growth factor removal from lt-NES cell lines grown in Shh/FGF8 for 14 days induced differentiation and appearance of cell clusters positive for FoxA2 and tyrosine hydroxylase (TH), with beta III-tubulin-positive neurons co-expressing Nurr1. In contrast, control cultures propagated in FGF2/EGF remained TH-negative. Abbreviations in upper right corners of the immunofluorescence photomicrographs denote lt-NES cell lines used. Scale bars: 50 µm.

Neural stem cell lines propagated in culture typically demonstrate restricted amenability to patterning cues [20]. We therefore investigated whether expanded lt-NES cells derived from iPSC can still be guided towards distinct neuronal phenotypes. To that end, we withdrew FGF2 and EGF and exposed the cells to sonic hedgehog (Shh) and fibroblast growth factor type 8 (FGF8), a well-known morphogen combination for the induction of ventral midbrain phenotypes [21]. After 7 days, individual cells co-expressing the ventral midbrain progenitor transcription factors LMX1A and FOXA2 were detected (Figure 3A). The proportion of FOXA2-expressing cells increased during the second week of treatment. A fraction of the cells also expressed Engrailed-1 (En1; Figure 3B). No expression of LMX1a or FOXA2 was detectable in the control population cultured in the presence of FGF2 and EGF (Figure 3B). After two weeks in Shh and FGF8, terminal differentiation was induced by growth factor withdrawal. Numerous neurons expressing tyrosine hydroxylase (TH) could subsequently be detected (Figure 3C–D). Some of the TH-positive neurons also showed nuclear expression of FOXA2. We also observed Nurr1-positive neurons upon Shh and FGF8 treatment. In contrast, cultures propagated under control conditions without morphogens contained only occasional TH-positive neurons and no FOXA2- or Nurr1-positive cells (Figure 3D). Together, these data demonstrate that lt-NES cells respond to morphogens by altering regional marker expression and neurotransmitter phenotype.

**Figure 4:**
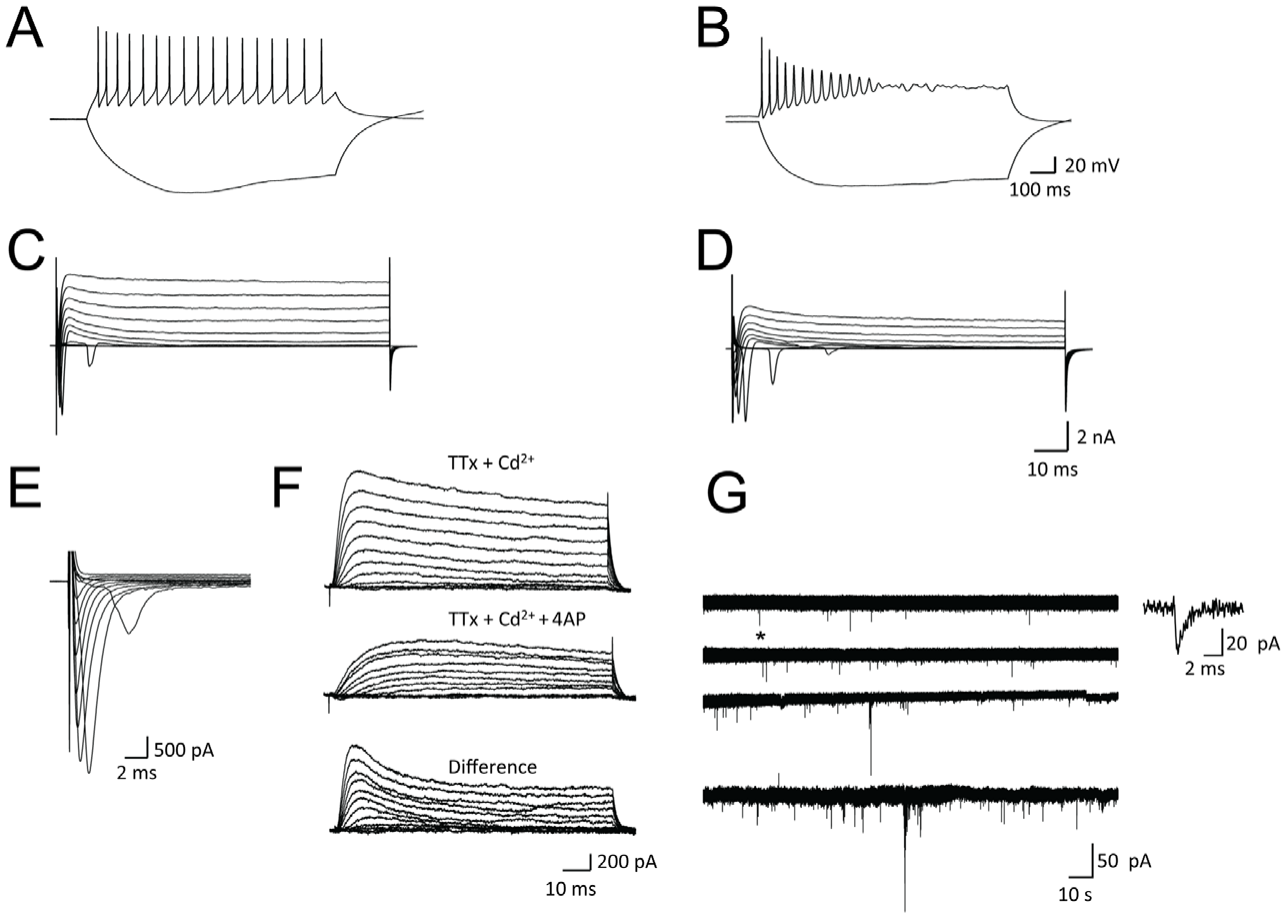
Lt-NES cells differentiate into functional neurons. Representative traces of membrane potentials in differentiated PKa (**A**) and PKc (**B**) lt-NES cells upon depolarization by current injection. Voltage-dependent membrane currents measured from the same neurons (**C–D**). Depolarizing voltage steps elicited voltage-dependent inward and outward currents. The fast and transient inward current could be isolated by use of intracellular Cs^+^, extra- and intracellular TEA and extracellular Cd^2+^ (**E**). PKc-derived neurons also expressed 4-AP-sensitive outward currents (**F**). (**G**) Representative traces of spontaneous synaptic responses in PKa (two upper traces) and PKc (two lower traces) neurons cultured on a monolayer of mouse astrocytes. A single postsynaptic current recording (asterisk) is shown in larger magnification.

We next asked whether neurons derived from iPSC-lt-NES cells are capable of generating electrically active neurons and forming functional neuronal networks engaging in synaptic transmission. Current-clamp measurements were performed on iPSC-derived neurons to elicit action potentials. Most of the neurons tested generated multiple action potentials upon current injection (Figure 4A–B). Voltage-dependent membrane currents were also observed in these neurons (Figure 4C–D). The fast and transient inward current was sensitive to the application of 300 nM TTx (a selective Na^+^ channel blocker) and could be isolated by use of intracellular Cs^+^, extra- and intracellular TEA, and extracellular Cd^2+^ (Figure 4E). Thus, the fast transient inward current observed in whole-cell voltage-clamp measurements of lt-NES cell-derived neurons is most probably carried by Na^+^ influx through voltage-dependent Na^+^ channels (I_Na_). Addition of 10 µM CdCl_2_ (which non-selectively blocks voltage-dependent Ca^2+^ channels) to the TTx-containing extracellular solution did not lead to further significant changes in the whole-cell current pattern (data not shown). In addition to TTx and CdCl_2_ we used 4-AP, which is known to preferentially block inactivating potassium channels (both A- and D-type). Application of 4-AP (5 mM) had a profound effect on the whole-cell outward current (Figure 4F). The remaining current after application of 4-AP exhibited a much slower onset, smaller amplitude, but no decline during the 90 ms lasting test pulse. The 4-AP-sensitive component of the outward current can be demonstrated by digital subtraction of the current sweeps obtained from measurements before and after drug application (Figure 4F). Thus, the cells express 4-AP sensitive K^+^ currents.

An essential feature of CNS neurons is their ability to form synapses. Spontaneous postsynaptic currents were observed in whole-cell voltage-clamp recordings performed at two months of differentiation in vitro (measured in PKa and PKb) indicating functional synaptic transmission (Figure 4G). These findings demonstrate that lt-NES cells are capable of generating electrophysiologically functional and synaptically connected neurons in vitro.

### Lt-NES cells from different sources share similar characteristics

**Figure 5:**
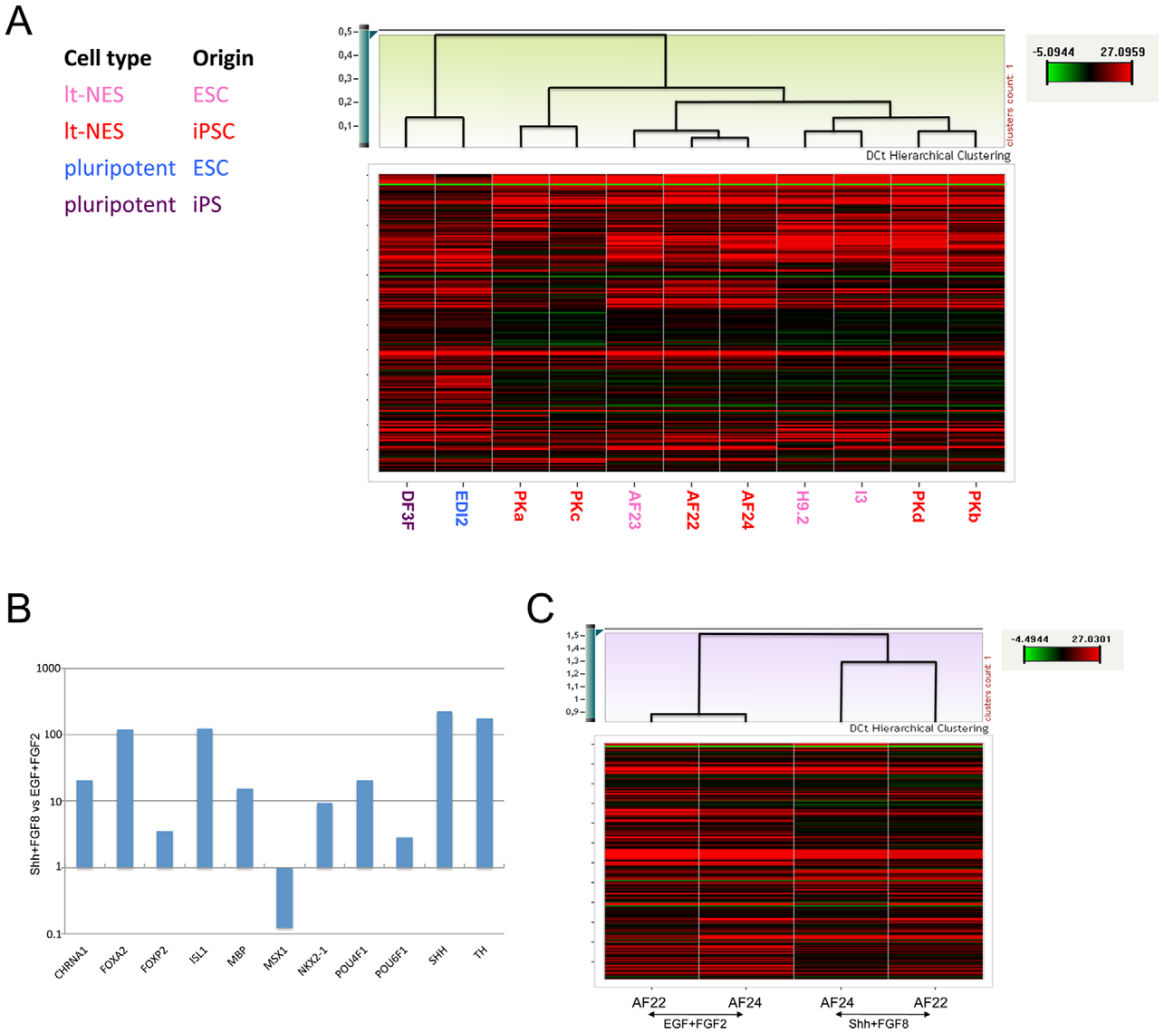
Lt-NES cell lines of various origins are similar to each other. (**A**) Heat map and hierarchical cluster analysis of TLDA expression data from 11 cell lines comprising 2 pluripotent and 9 lt-NES cell lines of different origin. The hierarchical cluster analysis (Pearson) using complete linkage based on delta CT gene expression values reveals two major clusters representing the two different cell types visualized. (**B**) Genes significantly up-regulated (corrected p-value threshold 0.05) in Shh- and FGF8-treated lt-NES cell lines compared to control culture conditions (EGF and FGF2). (**C**) Cluster analysis displays higher similarity between Shh/FGF8-treated lt-NES cell lines than to the parental lt-NES cell line still cultured in EGF and FGF2.

Previous studies have shown that the potential to generate somatic cell types including neuroectodermal cells can vary significantly between different hESC lines and between hESCs and iPSCs [5], [7]. To assess the variability between established lt-NES cell lines from different genetic backgrounds we designed a Taqman low-density real-time PCR array (TLDA) for a panel of genes known to be expressed in neural progenitor cells and the neural stem cell niche. We grouped the genes into different categories reflecting regional identity, neural rosette markers, transcription factors expressed in the ventricular zone or during differentiation, and cell cycle-associated genes (Figure S5). All 192 genes (including 18S and GAPDH for reference) were assayed in technical duplicates (aggregated using the median) (Table S1). Hierarchical clustering based on the delta CT gene expression values of nine lt-NES cell lines (three hESC-derived and six iPSC-derived) and two pluripotent cell lines (one hESC and one iPSC line) showed the two pluripotent cell lines together and distinct from the lt-NES cell lines (Figure 5A). Interestingly, inter-profile similarity between lt-NES cell lines appeared independent of whether they were hESC- or iPSC-derived (Figure 5A). Further to this observation, we observed no significant difference in expression between hESC- and iPSC-derived lt-NES cell lines for any of the 192 genes tested using the Limma moderated t-statistic [22] with corrected p-value threshold of 0.05.

Using the expression profiles we further evaluated the regional identity of nine different lt-NES cell lines based on the regional-specific markers present on the TLDA (Figure S6 A). In addition we performed end-point RT-PCR analysis of the rhombomere 4 specific marker KROX20, which was expressed in all nine lt-NES cell lines analyzed (Figure S6 B). These analyses further pinpointed the hindbrain phenotype of the lt-NES cell lines to a ventral anterior hindbrain identity.

TLDA analyses also confirmed significant expression changes upon exposure of lt-NES cells to patterning cues. Although the TLDAs are not designed to include all specification markers, several genes were up-regulated significantly (corrected p-value threshold 0.05) in four lt-NES cell lines treated with Shh and FGF8 compared to lt-NES cell lines grown in EGF and FGF2 (Figure 5B). In particular, lt-NES cells treated with Shh and FGF8 showed a markedly increased expression of ventral midbrain markers, including a more than 100-fold induction of FOXA2, SHH and TH (Figure 5B). Hierarchical cluster analysis revealed greater similarity between lt-NES cell lines treated with Shh and FGF8 than to their counterparts grown in EGF and FGF2 (Figure 5C; see also CT correlation plots in Figure S7).

### Lt-NES cells and radial glia-like NS cells are distinct neural stem cell populations

**Figure 6:**
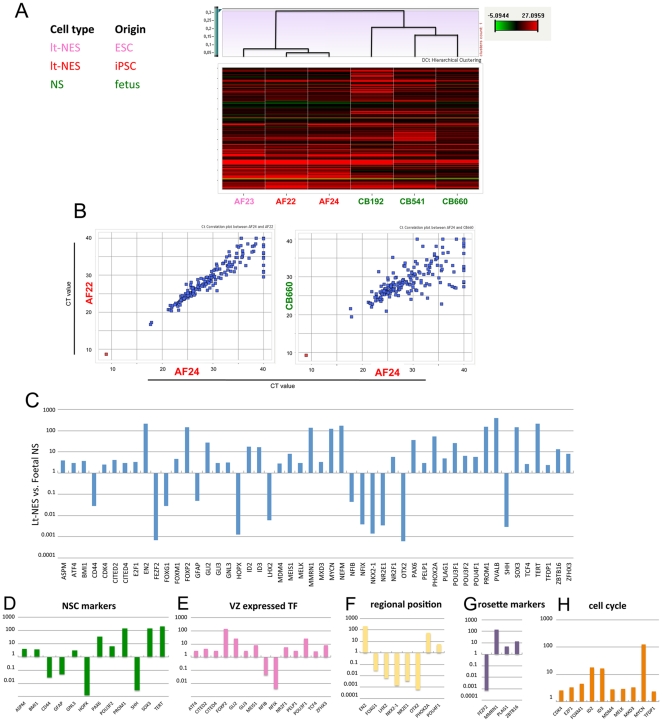
Gene expression analysis of lt-NES cells and fetal NS cells. (**A**) A total of six cell lines representing two cell types (lt-NES and fetal NS) of different origins were profiled using TLDAs. Hierarchical cluster visualization (Pearson) shows separation into two major clusters representing the two cell types analyzed. (**B**) Correlation plots based on CT gene expression values of the individual cell lines. The lt-NES cell lines AF24 and AF22 show a higher degree of correlation than the lt-NES cell line AF24 and the NS line CB660, although the latter carry the same genome (R = 0.95 vs. R = 0.76). Red dots represent the median aggregated CT expression value of the 18S endogenous control; blue dots indicate the median aggregated CT expression values of all other genes on the TLDA. (**C**) Relative levels of differently expressed genes (corrected p-value threshold 0.05) in 9 lt-NES cell lines and 3 fetal NS cell lines. (**D–H**) Panels depicting the expression of markers representing the categories neural stem cell (NSC) markers, ventricular zone (VZ) expressed transcription factors (TF), regional position, rosette genes, and cell cycle. Data in C–H are depicted at a Log10 scale with the mean expression in fetal NS cells set to 1. No significant differences in expression levels were detected in ESC- vs. iPSC-derived lt-NES cells.

Neural stem (NS) cell lines have previously been established from human fetal cortex (embryonic day 50–55) [13]. These NS cells resemble radial glia and, although maintained in adherent culture in similar media with FGF2 and EGF, their morphology and growth rate are different from those of lt-NES cells. We used the TLDAs to explore gene expression in NS and lt-NES cells. Hierarchical clustering showed that the fetal NS cell lines cluster together and are distinct from the lt-NES cell lines (Figure 6A). The lt-NES cell line AF24 originates from the reprogrammed fetal NS cell line CB660. Nonetheless, CT correlation plots revealed closer similarity to the unrelated lt-NES cell line AF22 than to its ancestor cell line (Figure 6B). To interrogate further the differences in the gene expression profiles between lt-NES and NS cells, we assessed differential gene expression across the delta CT gene expression profiles of 9 lt-NES and 3 fetal NS cell lines (Limma moderated t-statistic with corrected p-value threshold of 0.05). Many of the general neural stem cell markers such as SOX2 and FABP7 (BLBP) were expressed at similar levels (Table S1). However, 50 of the 192 genes showed significantly different expression between the two cell types (Figure 6C). We allocated the 50 differently expressed genes to categories reflecting biological function. i.e., “neural stem cell (NSC) markers”, “ventricular zone (VZ) expressed transcription factors (TF)”, “regional position”, “rosette genes” and “cell cycle” (Figure S5 and Figure 6D–H). Markers associated with neuroepithelial cells such as PAX6 and SOX3 were 20–100 fold up-regulated in lt-NES cells. Consistent with the flow cytometry data, PROMININ was highly expressed in lt-NES cells but lower in NS cells, which showed variable cell surface expression [12]. Transcripts associated with radial glia such as GFAP and CD44 were 20-fold up-regulated in fetal NS cells (Figure 6D). Differential expression of CD44 was also confirmed at the protein level and by flow cytometry (Figure S3). A similar observation was made for A2B5, which was expressed prominently in NS cells but only in a minor fraction of lt-NES cells (Figure S3). Transcription factors known to be prominently expressed in neurogenic regions in vivo, e.g., CITED2, 4, GLI2-3, MEIS1, NR2F1, and TCF4, were more highly expressed in lt-NES than in NS cells (Figure 6E). With respect to regional position, the cortex-derived fetal cells showed prominent expression of the anterior markers OTX2, LHX2, NKX2.1 and FOXG1, which were not detectable in lt-NES cells beyond passage 15 (Figure 6F). A reciprocal trend for the hindbrain markers GBX2, NKX6.1 or IRX3 was not observed at statistically significant levels in the overall analysis, probably due to the fact that two of the lt-NES cell lines (PKb and PKc) were investigated during earlier passages, prior to acquiring a consolidated posterior identity. Markers typically found in the early neuroepithelium such as MMRN1, PLAGL and ZBTB16 (PLZF) were strongly expressed in lt-NES cell lines but only weakly in radial glia-like NS cells (Figure 6G). We also detected 100-fold higher expression of telomerase (TERT) in lt-NES cells, comparable to levels in pluripotent stem cell lines. Moreover, lt-NES cells exhibited higher transcript levels for cell cycle-associated genes, including CDK4, E2F1, ID2-3, FOXM1, MDM4, MXD3 and MYCN (Figure 6H). This is consistent with the more rapid proliferation rate of lt-NES cells, which double every 24–30 hours compared to 2–3 days for fetal NS cells [13]. Together, these data indicate that lt-NES cells and NS cells are distinct neural stem cell types representing different developmental stages.

## Discussion

The advent of cell reprogramming and the prospect of analyzing the molecular pathogenesis of neurological disorders in iPSC-derived neurons and glia have created a demand for protocols that reproducibly and quantitatively yield defined neural populations. The identification and dissection of disease-relevant mechanisms will depend critically on the ability to compare consistently differentiated neural cell populations generated from genetically distinct pluripotent starting cells. Based on the findings in this study we suggest that lt-NES cells represent a valuable step in this direction.

The successful generation of lt-NES cells from iPSCs was independent of the parental cell type used for reprogramming, the factors used to induce pluripotency, culture conditions used for propagating the pluripotent cells and the variable propensity of the individual parental pluripotent stem cell lines to differentiate directly into neural cell types. Lt-NES cells show a highly characteristic morphology, differentiation potential and gene expression profile, which remain stable even after long-term propagation over more than 100 passages. Importantly, lt-NES cells exhibit a consistency that appears to override variations in initial differentiation efficiency, cell lines, and handling. Using a related approach to that applied here, Nemati et al. recently described the generation of an expandable neural precursor cell population from an iPS cell line [23]. However, their cultures showed only partial suppression of neuronal and glial differentiation, possibly due to the use of a serum substitute and/or of very high concentrations of FGF (100 ng/ml) in the culture media. Importantly, in our hands lt-NES cells invariably exhibited negligible expression of markers associated with differentiation during expansion culture.

Lt-NES cells display a characteristic gene expression signature independent of their hESC or hiPSC origin. They appear to represent a developmentally early cell population as reflected by their rosette-like growth pattern and marker expression. Consistent with this, lt-NES cells have a distinct transcription profile from the more radial glia-like fetal NS cells. Multiple cell cycle genes are upregulated in lt-NES cells, which is in accordance with their faster proliferation rate. Fetal NS cells express markers of radial glial cells (e.g. GFAP, CD44, A2B5), whereas lt-NES cells preferentially exhibit neuroepithelial and neural rosette markers (e.g. PAX6, PROM1, SOX3, MMRN1, PLZF), indicating that they represent an earlier stage of CNS development. Furthermore, anterior regional identity markers are maintained in the cortically derived fetal NS cells, whilst lt-NES cells adopt a hindbrain regional specification.

It is interesting that these two molecularly and developmentally distinct neural cell types can be propagated long term in nearly identical culture conditions. This is particular striking considering that FGF2 is generally considered to exert a dominant respecifying effect on neural progenitors in vitro [24], [25]. Remarkably, the early developmental stage of lt-NES cells appears to make them more amenable to regional re-specification. Under standard culture conditions, lt-NES cells have a rather well-defined regional identity compatible with a hindbrain phenotype. Despite this overall posteriorization, data from this and previous studies [11] suggest that these cells may be recruited into adjacent regional cell types, most provocatively into midbrain dopamine neurons. It will also be interesting to determine whether lt-NES cells may be differentiated in vitro into NS cells.

Considering their regional identity, lt-NES cells should constitute a particularly suitable tool for modeling a range of neurodegenerative diseases affecting neuronal and glial cells in midbrain, hindbrain and spinal cord. However, many pathophysiological pathways are based on common denominators such as oxidative stress response, axonal transport or synaptic plasticity, which are conserved among different regional subtypes. For that reason, we expect lt-NES cells to provide a useful tool for modeling diseases affecting many aspects of cellular neurobiology, and their advantages concerning highly reproducible derivation, easy handling, robust expandability and comparability might outweigh any limitations in regional plasticity. Notably, synaptic dysfunction is an early denominator of neurodegenerative and neurodevelopmental disease. The demonstration that lt-NES cells form spontaneously active neuronal networks provides prospects for employing them for functional studies of synaptic transmission in neurons with control and disease genotypes.

Once established, lt-NES cells are a robust population that can be subjected to repeated freeze-thawing, cryoconservation and automated spotting in multiwell formats. Based on their extensive self-renewal capacity, lt-NES cells are further amenable to various genetic modifications with high efficiency. This may be a stem cell reporter such as the Nestin-GFP employed here, or a neuron-specific selection marker as the one we previously used to generate virtually pure cultures of human neurons, which themselves can be subject to efficient cryopreservation [26]. Lt-NES cells may thus provide a consistent source of human neurons for pharmaceutical screening against defined molecular targets or for neurotoxicity studies. These properties make lt-NES cells potentially suitable for high throughput applications involving genetic and compound library screening. Biomedical applications might range from the analysis of disease mechanisms to refined drug discovery using disease-relevant human neurons and glia.

## Materials and Methods

### Ethics Statement

IPS cells used in this study were derived with written informed consent by the donors with respect to taking the samples and making the cell lines. IPS cell generation was approved by the Ethics Committee of the Medical Faculty, University of Bonn (permit # 275/08). The Institute of Reconstructive Neurobiology, University of Bonn has a license for neural differentiation of human ES cells (Robert Koch-Institute AZ 1710-79-1-4-1).

### Establishing and maintaining lt-NES cell lines

Culture conditions for the different human ESC and iPSC are summarized in Table 1. Cells were split every 3–6 days using collagenase (Invitrogen). Human ESC and iPSC were induced to differentiate as described [11]. Briefly, cells were dissociated into small aggregates using collagenase (Invitrogen) and plated on non-adhesive plastic in human ESC media (DMEM/F12 or knock-out DMEM, 0.1 mM NEAA, 0.1 mM beta-mercaptoethanol, 2 mM L-glutamine, 15% or 20% (KSR); all from Invitrogen) without FGF2 to induce differentiation. Media was changed every second to third day, and 5–7-day-old floating aggregates were plated on tissue culture plates coated with 0.1 mg/ml poly-L-ornithine (Sigma). Neural rosette structures started to emerge about one week after plating. Rosettes were carefully picked every second day between one and two weeks post plating. Picking was performed with a needle, and picked clusters were inspected under the microscope for purity before transfer to a non-adhesive culture plate containing DMEM/F12, 2 mM L-glutamine, 1.6 g/l glucose, 0.1 mg/ml Penicillin/Streptomycin and N2 supplement (1∶100; Invitrogen). After 2–5 days floating aggregates were dissociated in trypsin for 5–10 minutes. Trypsin activity was inhibited with trypsin inhibitor before cells were spun down for 5 minutes at 300 g. Media was carefully aspirated to avoid any remaining trypsin, and cells were plated onto poly-L-ornithine and 10 µg/ml laminin (Sigma) coated plates into the same media supplemented with 10 ng/ml FGF2, 10 ng/ml EGF (both from R&D systems) and B27 (1 µl/ml, Invitrogen). Cells were passaged at a ratio of 1∶3 every second to third day using trypsin. For cryopreservation cells were trypsinized, and around 2×10^6^ lt-NES cells were spun down (300 g) and resuspended in freeze media containing 10% DMSO and 90% culture media before controlled freezing at −1°C per minute. Cells were rapidly thawed at 37°C, resuspended in pre-warmed culture media, spun down for 5 minutes at 300 g and replated on poly-L-ornithine and laminin coated plates in the presence of FGF2, EGF and B27. Neuronal differentiation was induced as described earlier [11] by removing the growth factors FGF2 and EGF from the media and culturing the cells in a 1∶1 ratio mixture of Neurobasal media supplemented with B27 (1∶50, Invitrogen) and DMEM/F12 media supplemented with N2 (1∶100); 300 ng/ml cAMP was added to the differentiation media. The fate of the differentiated cells was quantitatively assessed by counting 250–650 cells in nine 20× microscope fields from 3–4 experiments. For induction of ventral midbrain phenotypes lt-NES cells were exposed to media containing Shh (100–500 ng/ml; R&D Systems) and FGF8 (200 ng/ml; R&D systems for AF lines and in-house made FGF8 500 ng/ml [27] for PK lines) for 14 days before being differentiated for 2–4 weeks.

### RNA preparation and RT-PCR

RNA was isolated using RNeasy columns from Qiagen, and cDNA was produced with Superscript III (Invitrogen). Relevant genes were amplified using Taq-polymerase with primers listed below.


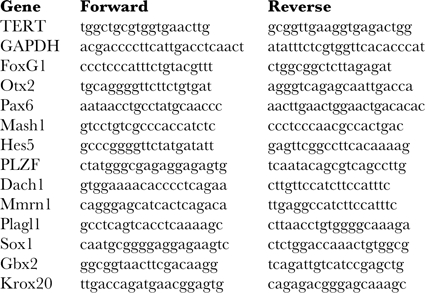


Alternatively, the cDNA was loaded onto a custom-designed TLDA card and technical duplicates were assayed using a 7900HT real-time PCR machine (Applied Biosystems). TLDA data were analyzed using StatMiner software (Integromics).

### Immunofluorescence

Cells were fixed in 4% paraformaldehyde for 10–20 minutes at room temperature, washed in PBS and incubated in blocking and permeabilization solution containing BSA (5% fraction V, Sigma), Triton X-100 (0.1%) and 10% fetal bovine calf serum (Sigma) for 1 hour at room temperature. For detection of GABA, 0,1% glutaraldehyde was added to the fixative. Primary antibodies were diluted in PBS or permeabilization solution and incubated with the cells at 4°C over night (antibodies and dilutions are listed below). Secondary antibodies conjugated to Alexa fluorophores (Invitrogen) were diluted 1∶1,000 and incubated for 1 hour at room temperature before washing and subsequent microscopic inspection.


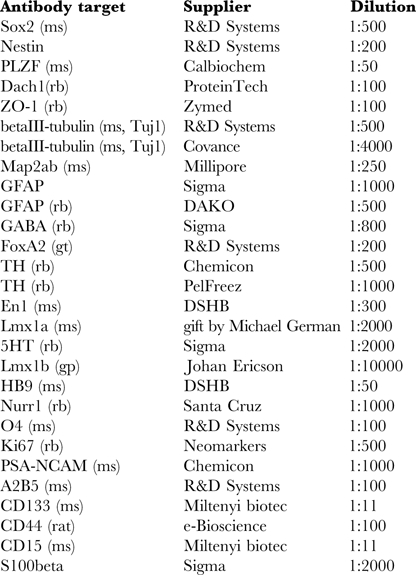


### Electrophysiological recordings

Lt-NES cells differentiated for two months on a mouse astrocyte feeder layer in vitro were selected for experiments. Whole cell current-clamp and voltage-clamp recording was carried out with an Axopatch-200B amplifier (Molecular Devices, USA) that was interfaced by an A/D-converter (Digidata 1440, Molecular Devices, USA) to a PC running PClamp software (Version 10, Molecular Devices). Pipette electrodes (GB150F-8P, Science products, Germany) were fabricated using a vertical puller (Narishige PC-10, Japan) and fire-polished (final tip resistances 2–4 MΩ). All recordings were performed at room temperature in a bath solution containing (in mM): 140 NaCl, 5 KCl, 2 CaCl_2_, 0.8 MgCl_2_, 10 Hepes and 10 glucose (pH 7.2; osmolality 310–320 mOsm). For most recordings of membrane potential or current, the patch pipette contained the following (in mM): 120 potassium gluconate (C_6_H_11_O_7_K), 20 KCl, 10 NaCl, 10 EGTA, 1 CaCl_2_, 4 Mg ATP, and 0.4 Na GTP, and 10 Hepes (pH 7.2, osmolality 280–290 mOsm). For some voltage-clamp recording, another pipette filling solution was used (in mM): 120 CsCl, 10 NaCl, 10 TEA-Cl, 10 EGTA, 1 CaCl_2_, 4 Mg ATP, and 0.4 Na GTP (pH 7.2, osmolality 280–290 mOsm).

### Clonal lines

Clonal lines were derived by deposition of single cells on a fibroblast feeder layer using a flow cytometer (Moflo, Dako Cytomation). Medium was changed every second day. After two weeks colonies were observed in around 10% of the wells. The colonies were trypsinized, removed from the feeder cells and plated onto poly-ornithine and laminin coated 96-well-plates for further expansion.

### Growth Kinetics

Pictures were taken every 15 minutes using the Incucyte system (Essen Instruments, USA) (see Movie S1), and growth was quantified on the basis of the area of the field occupied by cells.

### Nucleofection

To derive a lt-NES reporter line we used the second intron of the rat Nestin enhancer to drive GFP. We nucleofected 2×10^6^ AF22 cells mixed with 2 µg of the PiggyBac vector pGG131 [28] were the CAGDsRedIRESHygro was replaced by Nestin enhancer-driven GFP linked to Puromycin by an internal ribosome entry site (Nestin-GFP) together with 0.2 µg pBase (transposase) in 100 µl of Amaxa buffer. The mixture was added to Amaxa cuvettes before nucleofection using the T20 program (Amaxa). Cells were immediately added to pre-warmed expansion media and plated in a slot of a poly-ornithine and laminin coated 6-well-plate. Antibiotic selection was initiated in proliferating cells 24 hours post transfection (puromycin 1 µg/ml). Seven days later, colonies were dissociated and moved to other dishes for further expansion, assessment of GFP expression and cryopreservation.

### Bioinformatics

Correlation plots were based on CT values, and correlation heat-maps were colored according to Pearson correlation between sample profiles. Endogenous control values were calculated by median aggregation of 18S and GAPDH expression values and subtracted from the CT values in corresponding profiles to yield delta Ct (dCt) values. Hierarchical clustering was performed on dCt values and assessed inter-profile similarity using Pearson correlation and complete linkage. For each gene present, differential expression between profile groups was assessed using the Limma moderated t-statistic [22]. The resulting significance p-values were corrected for multiple testing using the Benjamini-Hochberg adjustment for false discovery. Differential gene expression between sample groups was deemed significant at a corrected p-value threshold of 0.05. All analyses were performed using the StatMiner software from Integromics.

### Flow cytometry

Cells were dissociated and detached with accutase, and living cells were stained with primary antibodies diluted in culture media at room temperature (RT) for 15 minutes before washed once and presented to the secondary antibody coupled to Alexa-488 (Molecular Probes, diluted 1∶1000) for 10 minutes at RT and washed again. TO-PRO-3 (Invitrogen) was used for live/dead discrimination, and pulse width was used to gate for single cells. Cell surface expression was analyzed using a flow cytometer (CYAN, Dako Cytomation), and data was analyzed using the FlowJo software (Tree Star Inc.). Both non-stained cells and cells stained without primary antibodies were used to set up the analyzing gates.

## Supporting Information

Figure S1 **iPSC used to derive lt-NES cell lines express human pluripotency markers.** The figure shows representative stainings of the two iPSC lines PkA (**A**) and DF3 (**B**).
(TIF)

Figure S2 **During proliferation, expression of beta III-tubulin is restricted to occasional neurons, which are due to spontaneous differentiation.** GFAP-positive astrocytes could not be detected under these conditions.
(TIF)

Figure S3 **Overlay presentations of flow cytometry data showing histograms for the expression of CD133 (A), CD15 (B), PSA-NCAM (C), A2B5 (D), and CD44 (E) in AF22 cells (A–E), AF23 cells (A), and CB660 cells (D, E).**
(TIF)

Figure S4 **Comparison of the glial differentiation potential of hESC- and iPSC-derived lt-NES.** Regardless of origin, lt-NES cells differentiate to beta III-tubulin positive neurons and glia cells positive for GFAP, S100beta, A2B5 or O4. The O4 staining of iPSC derived lt-NES cells is also provided in Figure 2. Depicted are cells differentiated for 6 weeks with exception of cells used for O4 detection, which were differentiated for 10 weeks. Regions of prominent glial differentiation were selected for the depicted images.
(TIF)

Figure S5 **List of genes represented on the Taqman low-density real-time PCR array.**
(TIF)

Figure S6 **Lt-NES cells display a distinct positional identity.** **A**: Evaluation of the regional identity of lt-NES cells based on the expression of region-specific transcription factors (compiled from Taqman low-density real-time PCR array (TLDA) data). Relative gene expression levels between the different lt-NES cell lines were determined by using the 2^−dCt^ method (normalizing to the housekeeping gene 18S). The Y-axis represents normalized relative gene expression (arbitrary units). **B**: End-point RT-PCR analysis showing expression of the rhombomere 4 specific gene KROX20 in nine different lt-NES cell lines.
(TIF)

Figure S7 **Correlation plots based on CT values of individual lt-NES cell lines treated with Shh and FGF8 vs. the same cell lines grown in EGF and FGF2.** There is higher correlation between different cell lines treated with the same growth factors than between the same cell line treated with different growth factors. Red dots represent the CT value of the 18S endogenous control and blue dots the CT values of all other genes on the TLDAs.
(TIF)

Table S1 **Expression profile of human lt-NES cells, fetal NS cells, hES cells and hiPS cells.** Numbers represent CT values. Undetermined expression is set to CT = 40.
(PDF)

Movie S1 **During expansion lt-NES cells self-organize in rosette-like structures.** Cell line AF22 (passage 21) was plated at low density and followed for 59 hours. Pictures were taken every 15 minutes.
(MP4)
