# Supplementary figures and images for: Capture of Neuroepithelial-Like Stem Cells from Pluripotent Stem Cells Provides a Versatile System for In Vitro Production of Human Neurons
Source: PLoS One. 2012 Jan 17;7(1):e29597. doi: 10.1371/journal.pone.0029597 (PMC3260177; doi:10.1371/journal.pone.0029597)

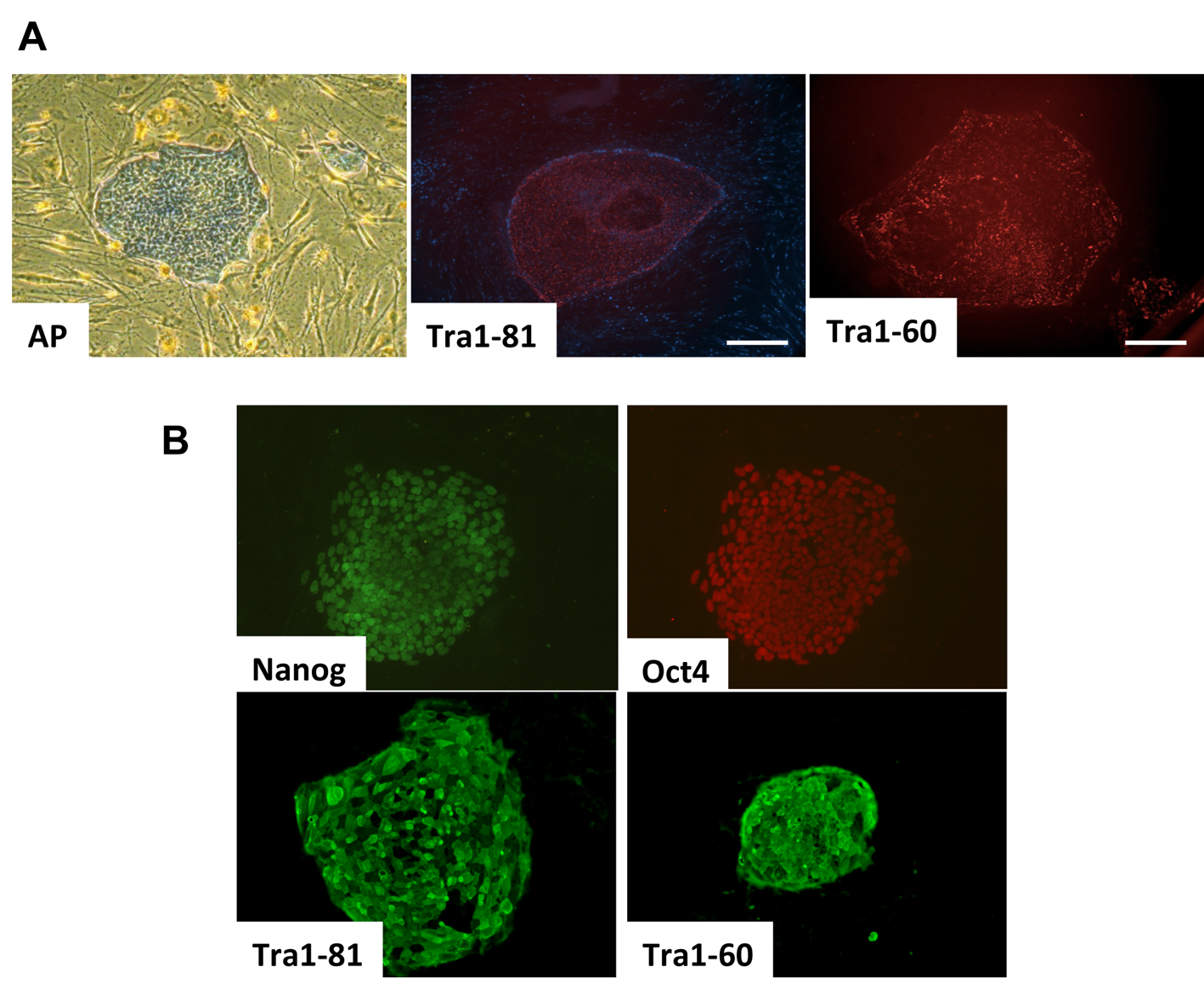

Supplement: Figure S1 — iPSC used to derive lt-NES cell lines express human pluripotency markers. The figure shows representative stainings of the two iPSC lines PkA (A) and DF3 (B). (TIF) [file pone.0029597.s001.tif]

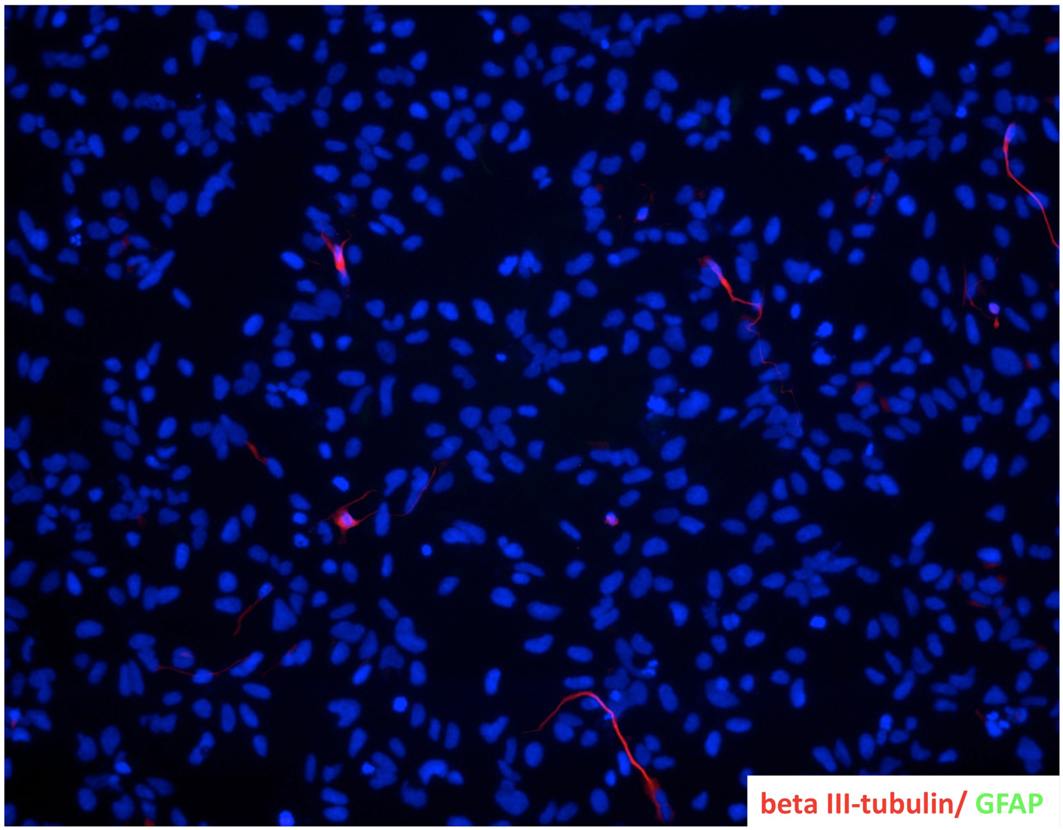

Supplement: Figure S2 — During proliferation, expression of beta III-tubulin is restricted to occasional neurons, which are due to spontaneous differentiation. GFAP-positive astrocytes could not be detected under these conditions. (TIF) [file pone.0029597.s002.tif]

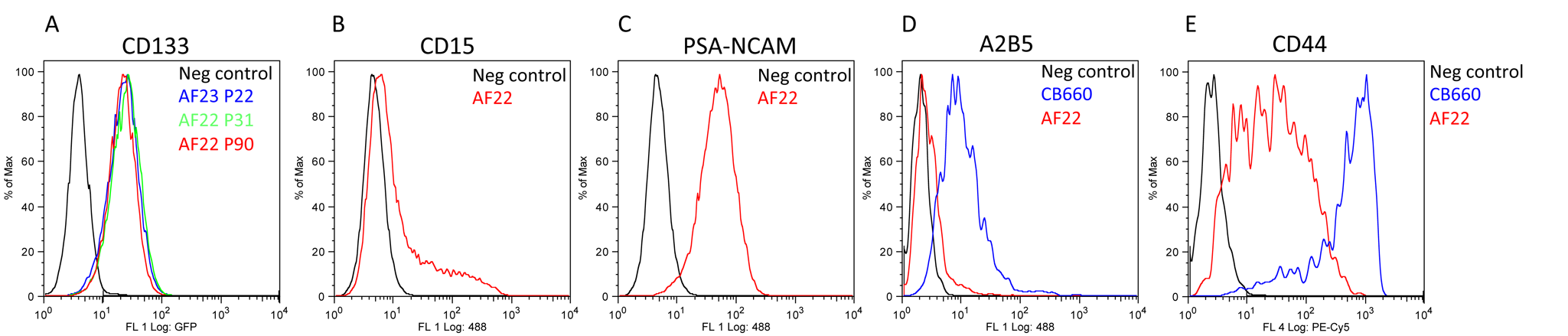

Supplement: Figure S3 — Overlay presentations of flow cytometry data showing histograms for the expression of CD133 (A), CD15 (B), PSA-NCAM (C), A2B5 (D), and CD44 (E) in AF22 cells (A–E), AF23 cells (A), and CB660 cells (D, E). (TIF) [file pone.0029597.s003.tif]

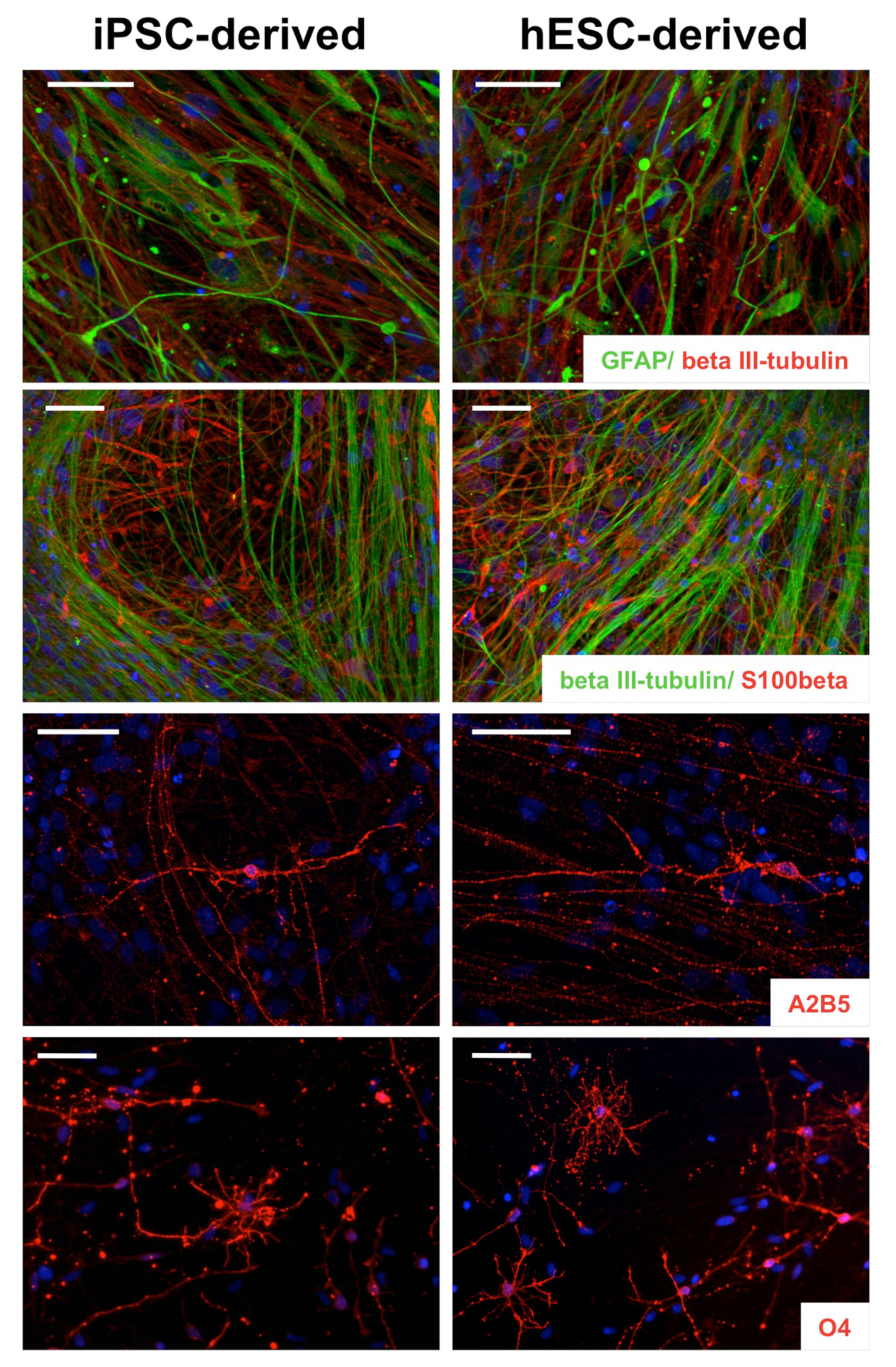

Supplement: Figure S4 — Comparison of the glial differentiation potential of hESC- and iPSC-derived lt-NES. Regardless of origin, lt-NES cells differentiate to beta III-tubulin positive neurons and glia cells positive for GFAP, S100beta, A2B5 or O4. The O4 staining of iPSC derived lt-NES cells is also provided in Figure 2. Depicted are cells differentiated for 6 weeks with exception of cells used for O4 detection, which were differentiated for 10 weeks. Regions of prominent glial differentiation were selected for the depicted images. (TIF) [file pone.0029597.s004.tif]

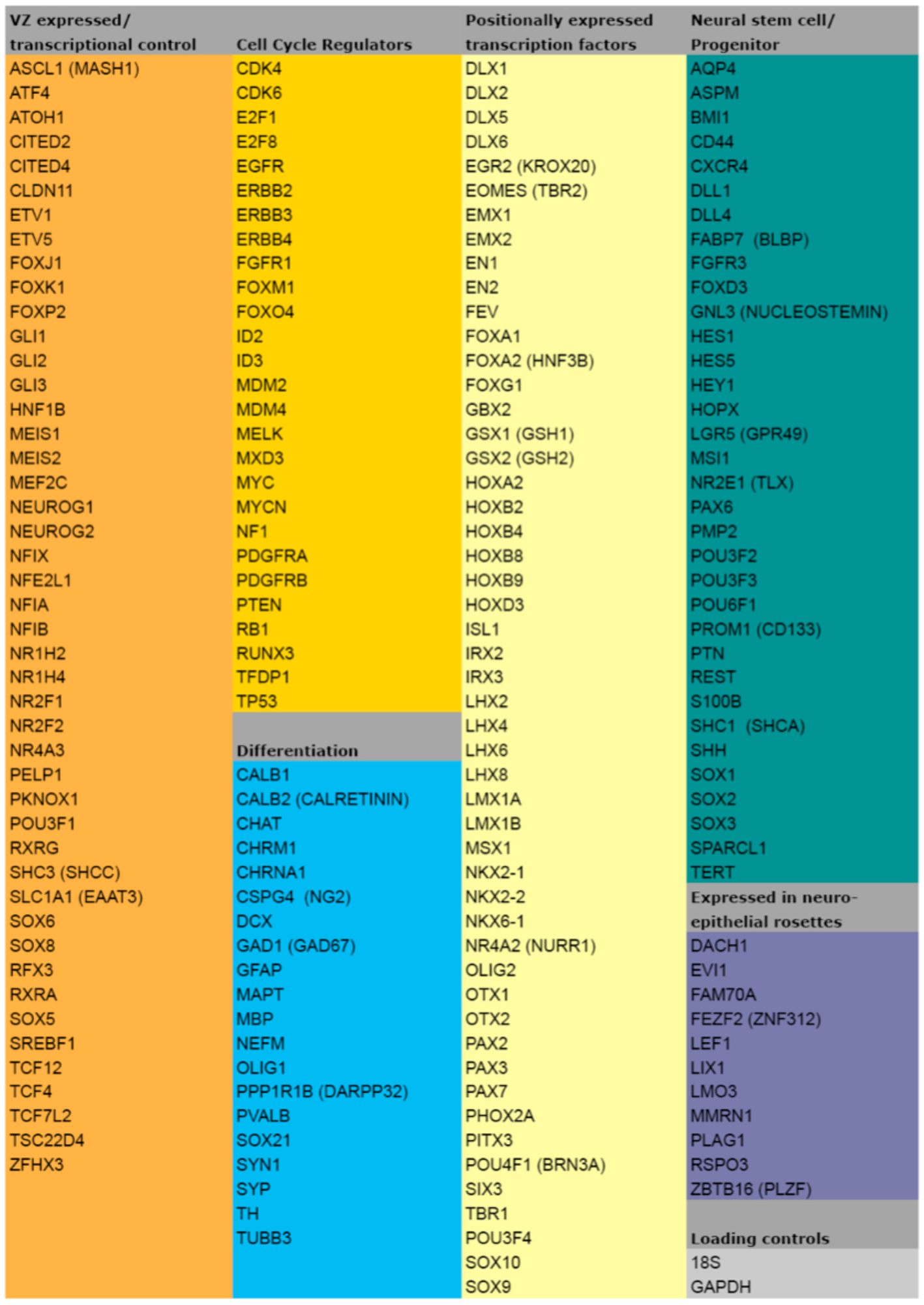

Supplement: Figure S5 — List of genes represented on the Taqman low-density real-time PCR array. (TIF) [file pone.0029597.s005.tif]

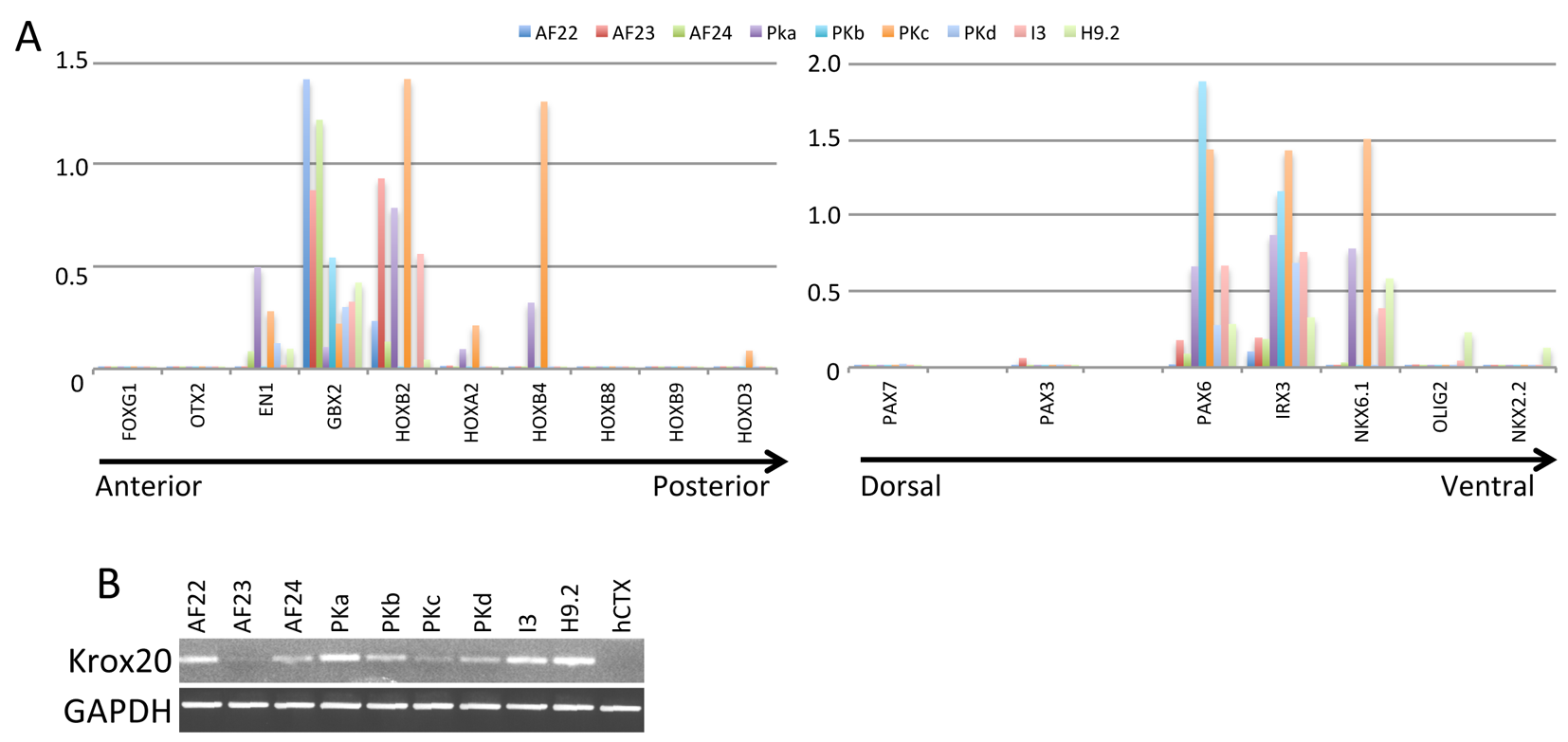

Supplement: Figure S6 — Lt-NES cells display a distinct positional identity. A: Evaluation of the regional identity of lt-NES cells based on the expression of region-specific transcription factors (compiled from Taqman low-density real-time PCR array (TLDA) data). Relative gene expression levels between the different lt-NES cell lines were determined by using the 2−dCt method (normalizing to the housekeeping gene 18S). The Y-axis represents normalized relative gene expression (arbitrary units). B: End-point RT-PCR analysis showing expression of the rhombomere 4 specific gene KROX20 in nine different lt-NES cell lines. (TIF) [file pone.0029597.s006.tif]

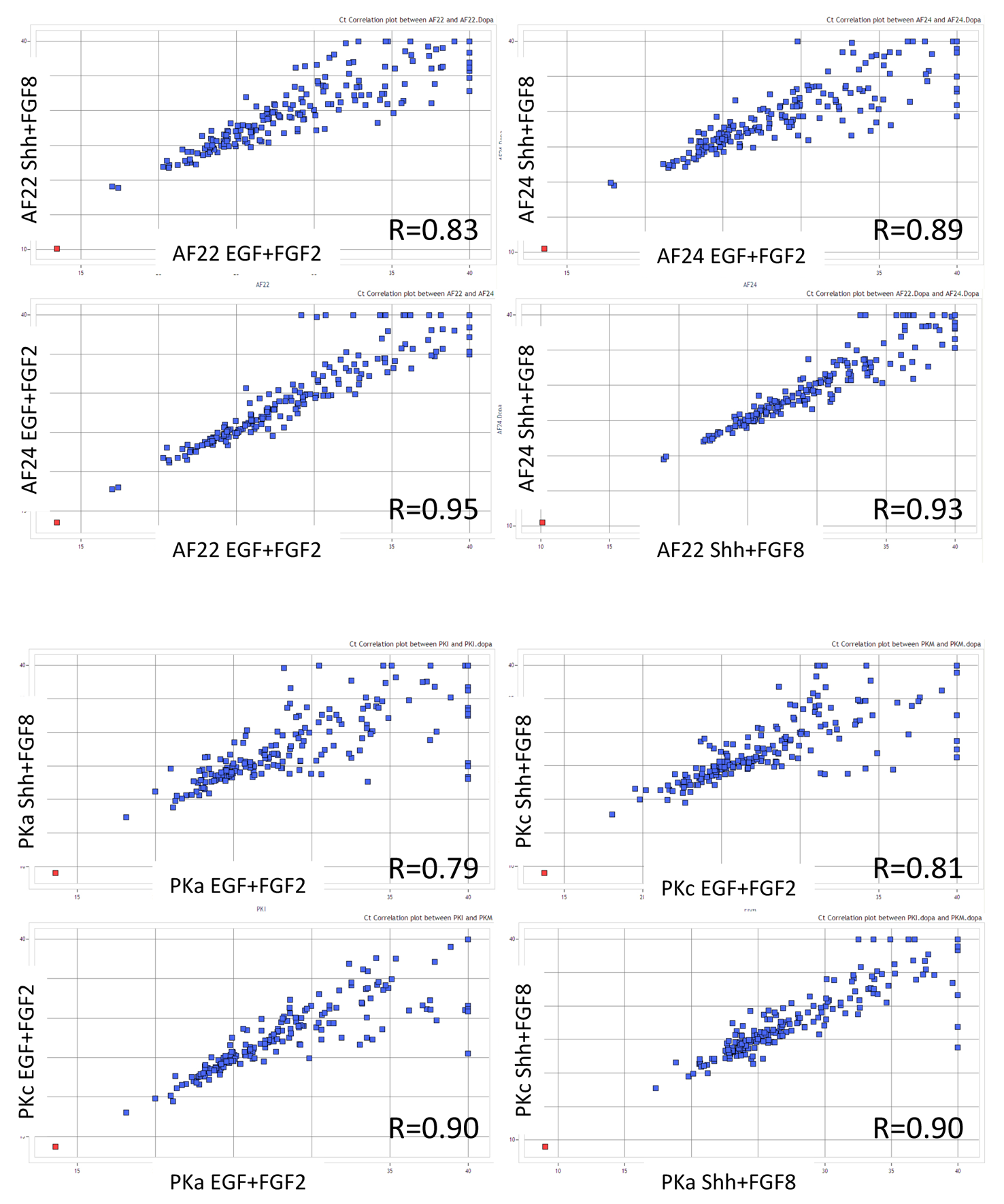

Supplement: Figure S7 — Correlation plots based on CT values of individual lt-NES cell lines treated with Shh and FGF8 vs. the same cell lines grown in EGF and FGF2. There is higher correlation between different cell lines treated with the same growth factors than between the same cell line treated with different growth factors. Red dots represent the CT value of the 18S endogenous control and blue dots the CT values of all other genes on the TLDAs. (TIF) [file pone.0029597.s007.tif]
