# Supplementary material for: Capture of Neuroepithelial-Like Stem Cells from Pluripotent Stem Cells Provides a Versatile System for In Vitro Production of Human Neurons
Source: PLoS One. 2012 Jan 17;7(1):e29597. doi: 10.1371/journal.pone.0029597 (PMC3260177; doi:10.1371/journal.pone.0029597)
Supplement: Table S1 — Expression profile of human lt-NES cells, fetal NS cells, hES cells and hiPS cells. Numbers represent CT values. Undetermined expression is set to CT = 40. (PDF) [file pone.0029597.s008.pdf]

Gene	Edi-2	Edi-2	DF-3F	DF-3F	CB541	CB541	CB660	CB660	CB192	CB192	AF22	AF22	AF23	AF23	AF24	AF24	PKb	PKb	PKc	PKc	Pka	Pka	PKd	PKd	I3	I3	H9.2	H9.2
18S-Hs99999901_s1	8.904	8.952	8.868	9.036	10.08	10.27	9.138	9.075	8.812	8.895	8.583	8.434	8.46	8.522	8.816	8.998	9.754	9.92	10.14	9.887	9.364	9.437	9.422	8.912	10.56	10.24	9.466	9.72
AQP4-Hs00242342_m1	34.19	33.3	31	30.6	33.7	33.57	30.8	30.28	31.6	31.66	31.4	31.79	30.53	30.71	30.76	30.18	32.3	31.94	29.79	29.79	32.52	31.72	31.49	31.92	37.04	35.13	33.68	33.93
ASCL1-Hs00269932_m1	32.32	33.13	27.21	27.31	26.82	26.81	27.31	27.3	32.51	32.33	30.14	30.22	28.09	28.19	30.1	29.91	25.53	25.72	22.62	22.64	22.77	22.88	27.71	27.99	26.61	26.6	26.99	27.11
ASPM-Hs00411505_m1	25.15	25.04	24.25	24.41	27.88	27.67	25.76	25.56	25.41	25.61	23	23.21	22.83	23.12	23.77	23.94	23.55	23.61	24.38	24.66	24.28	24.29	23.35	23.25	23.99	24.18	22.67	22.56
ATF4-Hs00909568_g1	22.12	21.98	21.22	21.28	24.88	24.67	23.27	23.39	22.64	22.73	20.69	20.72	21.36	21.04	21.27	21.22	21.23	21.43	21.21	21.12	21.39	21.45	21.44	20.9	21.57	21.86	21.51	21.77
ATOH1-Hs00245453_s1	30.41	30.67	28.85	28.96	34.87	36.04	40	36.18	40	40	35.08	34.6	36.82	34	34.63	34.55	34.34	35.94	35.42	40	33.89	35.64	35.43	34	40	36.28	35.3	40
BMI1-Hs00180411_m1	26.8	26.75	27.18	27.33	28.72	28.64	27.7	27.84	27.32	27.33	25.32	25.35	24.82	24.63	25.66	25.35	25.22	25.37	25.08	25.26	23.79	25.42	25.14	25.38	25.9	26.01	25.72	25.72
CALB1-Hs00191821_m1	26.16	26.06	22.41	22.21	25.84	25.91	24.07	24.01	21.48	21.28	23.49	23.45	26.24	26.31	23.97	23.84	25.41	25.34	25.07	25.1	30.31	30.09	28.5	28.19	28.68	28.75	29.03	28.62
CALB2-Hs00418693_m1	29.23	30.68	29.66	29.79	40	40	33.97	32.97	35.58	35.98	25.98	25.73	32.78	32.63	30.67	31.02	35.14	35.49	30.66	30.35	30.82	30.66	35.1	35.34	33.12	40	34.4	33.52
CD44-Hs00153304_m1	24.67	24.55	26.6	26.4	24.49	24.58	26.12	26.02	21.4	21.32	26.03	25.94	26.89	26.65	28.92	29	29.14	28.99	26.83	26.79	29.39	29.37	31.71	31.31	27.72	27.71	29.07	28.9
18S-Hs99999901_s1	9.074	9.055	9.155	9.049	10.46	10.33	9.514	9.413	9.187	8.994	8.795	8.861	8.59	8.591	9.148	9.051	9.839	9.931	9.987	10.06	9.298	9.424	9.534	9.391	10.75	10.63	9.784	9.408
CDK4-Hs00364847_m1	22.67	22.69	22.31	22.31	24.43	24.41	23.11	23.13	22.81	22.91	20.68	20.68	20.52	20.64	21.51	21.66	21.55	21.5	21.68	21.82	21.95	22.05	21.03	21.26	21.9	21.92	20.93	20.8
CDK6-Hs00608037_m1	24.81	24.89	25.3	25.02	24.77	24.77	24.76	24.75	24.25	24.11	22.29	22.07	23.21	22.99	22.99	22.73	25.26	25.03	22.32	22.06	22.5	22.49	23.23	23.09	25.16	24.98	25.12	25.06
CHAT-Hs00252848_m1	40	40	35.45	40	40	40	35.9	35.43	40	40	33.44	33.81	34.39	34.95	34.09	34.42	34.2	34.41	28.33	28.28	27.86	28.25	34.04	34.57	35.64	36.9	40	37.26
CHRM1-Hs00912795_m1	37.24	35.45	33.94	35.16	40	40	40	40	40	40	40	40	40	40	40	40	36.58	40	40	40	40	40	40	40	40	40	40	40
CHRNA1-Hs00175578_m1	33.97	33.35	30.8	31.03	30.06	29.91	28.19	28.17	29.92	29.58	30.14	30.03	32.99	34.13	32.76	32.64	31.69	31.44	30.01	29.87	26.91	26.96	32.78	32.76	34.5	34.52	34.49	34.59
CITED2-Hs01897804_s1	26.41	26.34	25.43	25.4	28.89	28.9	26.7	26.72	27.87	27.98	24.46	24.53	24.35	24.05	24.87	24.99	24.06	24.52	24.04	24.08	24.44	24.54	26.26	26.15	26.18	26.23	26.09	25.99
CITED4-Hs00388363_s1	26.76	26.68	26.07	25.91	30.27	29.57	28.14	27.95	26.77	26.62	26.19	25.9	25.84	25.35	26.39	26.09	25.61	25.46	26.33	26.05	26.45	26.27	25.55	25.52	26.18	25.96	25.22	25
CLDN11-Hs00194440_m1	28.18	27.97	27.33	27.33	40	36	31.48	31.25	30.11	29.91	29.38	29.34	29.69	29.64	31.26	31.12	33.03	33.11	33.3	34.44	34.6	35.56	34.24	33.39	35.68	40	35.7	34.85
CSPG4-Hs00426981_m1	29.27	29.67	28.33	28.43	32	32.52	29.2	29.13	28.33	28.24	28.78	28.62	29.96	30.12	29.32	29.92	30.56	30.91	30.27	29.66	29.73	29.64	32.69	35.79	29.14	29.23	29.56	29.7
CXCR4-Hs02330069_s1	31.24	31.61	34.26	33.62	40	40	32.63	32.41	30.79	30.63	33.34	33.73	32.02	32.92	33.08	33.57	30.72	31.02	27.13	27.51	27.63	27.53	27.44	27.49	30.78	30.61	28.03	28.08
DACH1-Hs00189301_m1	29.21	29.03	29.82	29.79	32.47	31.91	28.42	28.33	25.88	25.78	27.33	27.18	26.73	26.91	27.26	26.97	24.46	24.26	23.97	23.74	24.44	24.34	23.49	23.49	24.55	24.62	24.67	24.54
DCX-Hs01035496_m1	30.27	30.5	29.25	29.25	33.98	35.31	29.38	29.85	40	40	31.19	31.78	29.22	29.07	30.7	30.93	26.42	26.92	24.29	24.32	25.55	25.33	26.76	26.74	27.13	27.27	26.51	26.44
DLL1-Hs01011325_g1	30.59	30.57	27.21	27.71	29.2	29.22	28.77	28.75	29.28	29.16	29.31	29.44	26.33	26.45	28.71	28.75	24.45	24.66	23.59	23.48	23.97	24.02	24.56	24.4	25.45	25.46	25.14	24.88
DLL4-Hs00184092_m1	34.97	35.11	32.06	32.22	35.17	34.47	35.93	34.48	34.33	33.96	35.57	34.08	40	34.79	34.67	35.03	29.79	29.56	28.75	28.79	28.06	27.91	30.37	30.25	32.96	32.36	32.59	33.23
DLX1-Hs00698288_m1	33.5	33.97	31.42	31.15	27.93	27.81	26.4	26.32	28.45	28.44	28.36	28.28	31.74	31.64	29.61	29.74	28.08	28.05	23.77	23.82	23.26	23.59	32.69	33.38	33.89	32.49	31.99	31.62
DLX2-Hs00269993_m1	33.06	34.15	30.7	30.72	29.88	30	26.5	26.62	30.33	30.35	28.8	28.88	33.19	33.19	30.65	30.42	28.02	28.06	24.01	23.98	23.28	23.22	30.47	30.84	34.22	34.62	32.32	32.87
DLX5-Hs00193291_m1	30.43	30.22	32.59	32.08	35.36	35.3	28.37	28.63	35.86	40	32.6	34.42	40	40	35.92	34.9	30.98	30.71	28.69	28.68	25.97	26.09	40	40	40	40	40	40
DLX6-Hs00231999_m1	36.08	35.11	34.72	34.45	40	37.07	31.44	31.15	40	40	37.08	35.94	40	40	40	36.2	32.99	34.19	30.76	30.63	29.12	29.2	37.83	40	40	40	40	40
E2F1-Hs00153451_m1	25.68	25.62	25.19	25.17	27.15	27.14	25.83	25.85	25.08	25.03	23.23	23.26	22.8	22.86	23.67	23.73	23.79	23.74	23.76	23.75	24.17	24.11	23.02	22.99	24.04	24.1	23.14	23.03
E2F8-Hs00226635_m1	28.62	28.84	28.46	28.42	31.23	31.51	29.36	29.27	28.51	28.55	27.67	27.54	27.51	27.93	28	28.24	28.89	29.29	29.15	29.17	29.51	29.5	26.93	26.84	30.37	30.42	29.02	29.06
EGFR-Hs01076092_m1	28.35	28.2	27.85	28.4	29.06	29.35	26.89	26.71	25.48	25.74	26.48	26.63	25.97	25.64	25.57	25.91	25.62	25.75	28.81	28.94	27.18	27.13	24.85	25.08	26.15	26.19	24.97	24.73
EGR2-Hs00166165_m1	32.41	31.63	29.05	28.96	29.67	29.73	28.15	28.1	27.4	27.26	29.59	29.44	29.68	29.2	29.05	28.73	26.3	26.27	28.77	28.72	25.75	25.69	22.23	22.32	26.13	26.24	24.81	24.62
EMX1-Hs00417957_m1	31.26	31.51	30.7	31.26	40	40	40	40	40	40	40	40	40	35.66	40	40	40	40	40	40	40	40	40	40	40	40	40	40
EMX2-Hs00244574_m1	40	40	37.21	36.66	40	40	36.35	36.12	40	40	31.38	30.68	30.05	29.64	40	40	36.5	35.35	32.5	31.59	40	40	40	40	26.95	27.32	40	40
EN1-Hs00154977_m1	36.7	40	30.73	30.7	25.9	25.91	27.19	27.31	40	40	28.11	28.15	31	31.19	25.73	25.73	29.82	29.86	25.16	25.09	23.7	23.69	25.41	25.49	29.29	29.42	26.2	26.25
EN2-Hs00171321_m1	36.36	40	29.93	29.6	27.9	27.82	31.58	31.16	40	37.32	22.63	22.61	21.56	21.36	23.45	23.14	24.87	24.8	25.97	25.72	26.44	26.3	22.45	22.46	25.67	25.38	24.81	24.62
EOMES-Hs00172872_m1	25.05	24.83	30.99	31.54	40	40	40	36.13	36.34	34.16	40	40	34.01	32.79	40	40	36.98	36.19	34.57	33.31	34.54	35.97	35.45	36.28	40	40	36.06	36.6

Gene	Edi-2	Edi-2	DF-3F	DF-3F	CB541	CB541	CB660	CB660	CB192	CB192	AF22	AF22	AF23	AF23	AF24	AF24	PKb	PKb	PKc	PKc	Pka	Pka	PKd	PKd	I3	I3	H9.2	H9.2
ERBB2-Hs01001596_g1	22.15	22.19	21.77	21.78	25.23	25.32	24.34	24.49	25.26	25.28	22.96	23	22.29	22.27	23.54	23.53	23.08	23.19	23.59	23.52	23.17	23.32	22.04	22.18	23.74	23.75	22.1	22.17
ERBB3-Hs00951455_m1	26.15	26.36	25.37	25.3	34.24	36.62	30.57	30.87	34.2	34.56	29.44	29.72	25.76	25.83	29.88	30.08	29.56	29.53	28.83	29.07	29.34	29.25	32.61	33.33	29.42	29.41	29.62	29.17
ERBB4-Hs00171783_m1	35.05	34.79	34.11	34.4	31.81	32.11	27.1	27.21	33.35	34.45	29.2	29.16	27.48	27.58	27.78	27.6	26.18	26.48	27.87	27.99	28.97	28.89	25.97	25.95	28.59	28.37	27.98	28.13
ETV1-Hs00290469_s1	26.7	26.44	25.35	25.11	26.84	26.69	26.43	26.5	24.8	24.67	24.83	24.67	25.08	24.89	25.76	25.42	24.85	24.72	23.75	23.19	24.46	24.18	26.79	26.68	25.57	25.29	26.21	25.9
ETV5-Hs00231790_m1	26.62	26.66	27.62	27.69	26.64	26.55	26.15	26.2	25.96	26.01	25.25	25.37	24.55	24.78	25.58	25.66	25.71	25.82	25.42	25.51	25.15	25.3	25.51	25.62	25.5	25.77	25.14	25.27
EVI1-Hs00602795_m1	33.17	32.89	33.28	33.38	36.35	36.49	33.47	33.71	36.2	40	31.16	30.96	31.13	31.07	33.41	33.71	32.15	32.62	29.98	29.94	28.7	28.52	33.82	34.19	30.31	30.59	32.69	32.97
FABP7-Hs00361426_m1	29.42	29.38	26.86	26.83	23.13	23	23.65	23.5	21.84	21.78	24.4	24.41	24.43	24.35	23.79	23.6	21.9	21.55	19.93	19.75	20.05	19.96	22.36	22.38	23.43	23.1	24.32	24.48
FAM70A-Hs00215705_m1	31.53	31.85	31.92	32.23	32.76	32.95	30.79	30.57	26.84	27.04	33.48	33.13	31.07	31.57	32.25	32.11	30.47	29.98	30.44	30.74	33.51	35.1	28.62	28.61	40	40	40	40
FEV-Hs00232733_m1	40	40	40	40	40	40	40	40	40	40	40	40	40	40	40	40	40	40	29.55	29.53	31.55	31.6	40	40	35.85	35.45	40	40
FEZF2-Hs00375188_m1	32.83	33.5	28.1	28	27.59	27.79	24.41	24.42	26.98	26.93	32.3	33.01	30.1	29.68	33.14	32.59	40	40	31.36	31.88	40	40	40	40	35.41	40	40	40
FGFR1-Hs00259959_s1	27.27	26.97	26.66	26.76	29.67	29.99	28.58	28.7	28.6	28.63	27.19	27.26	27.04	26.8	27.65	27.36	26.57	26.45	27.9	27.93	27.63	27.57	26.28	26.5	27.12	27.09	26.86	26.82
FGFR3-Hs00997393_g1	25.14	24.96	25.19	25.01	29.14	29.42	26.36	26.41	30.56	30.59	26.63	26.84	25.61	25.5	26.1	26.19	24	24.06	24.96	24.92	26.33	26.41	23.25	23.64	24.73	24.68	24.14	24.25
FOXA1-Hs00270129_m1	32.33	32.34	35.33	35.78	33.94	35.79	32.85	32.73	36.98	36.63	40	40	36.72	36.84	40	40	35.89	35.49	40	40	40	40	34.3	34.19	40	40	33.34	33.21
FOXA2-Hs00232764_m1	27.45	27.01	32.76	34.51	40	40	40	40	40	40	36.44	40	40	40	40	40	40	40	40	40	40	40	40	40	40	40	40	40
FOXD3-Hs00255287_s1	26.59	26.83	25.55	25.53	40	40	32.7	33.42	35.8	40	40	35.72	33.13	32.48	33.42	36.19	34.41	40	27.58	27.72	33.7	33.42	40	40	35.93	36.19	35.66	34.99
FOXG1-Hs00702391_s1	31.7	31.95	33.48	33.05	33.5	33.64	27.5	27.49	32.7	32.52	36.96	35.56	37.21	35.24	35.5	40	33.22	34.43	31	31.53	40	35.11	35.94	35.49	35.75	36.81	35.51	35.24
FOXJ1-Hs00230964_m1	30.46	30.41	31.1	30.93	30.67	30.79	29.15	29.22	33.25	33.85	29.84	30.15	27.08	26.97	29.72	29.51	28.89	28.63	27.97	28.57	28.29	28.21	27.05	27.21	30.48	30.67	28.36	28.25
FOXK1-Hs01595620_m1	27.44	27.56	27.33	27.53	26.98	26.68	25.83	25.41	26.74	26.5	25.53	25.11	25.36	25.47	25.45	25.58	25.57	25.99	25.34	25.66	25.28	25.18	25.25	25.78	25.36	25.61	25.76	25.81
FOXM1-Hs00153543_m1	24.79	24.86	24.91	24.99	28.61	28.64	26.83	26.78	25.83	25.8	23.73	23.66	23.49	23.34	24.46	24.46	23.96	23.87	24.43	24.41	24.71	24.74	23.63	23.59	24.96	25.08	23.94	23.91
FOXO4-Hs00172973_m1	28.18	28.05	27.67	27.41	32.13	32.04	30.18	30.44	31.6	31.35	30.06	30.22	27.91	27.66	29.95	30.28	28.87	28.72	29.49	29.27	29.69	29.42	29.78	29.81	29.57	29.58	29.11	28.8
FOXP2-Hs00362817_m1	31.93	32.38	32.31	31.66	34.18	34.48	30	29.8	40	40	26.41	26.35	25.56	25.65	26.78	26.68	26.32	26.08	27.32	27.07	27.43	27.41	27.03	27.02	27.65	27.5	27.57	27.52
GAD1-Hs01065893_m1	25.28	25.29	28.27	28.06	30.82	30.75	27.98	27.93	27.02	27.1	35.66	33.57	40	34.38	40	34.34	30.82	30.82	26.75	26.71	24.81	25.08	30.41	30.51	27.16	27.43	26.33	26.31
GAPDH-Hs01922876_u1	18.6	18.62	17.72	17.77	20.12	20.19	19.47	19.46	18.58	18.49	17.21	17.29	17.22	17.28	17.88	18.03	17.64	17.65	17.89	18.05	18.07	18.07	18.27	18.16	18.43	18.65	18.18	18.4
GBX2-Hs00230965_m1	28.45	28.51	26.72	26.72	29.7	29.79	26.11	26.04	24.25	24.27	21.39	21.19	21.94	22	21.93	21.89	24.18	23.82	25.62	25.34	25.93	25.88	24.13	24.23	25.26	25.32	24.07	24.17
GFAP-Hs00909238_g1	31.57	32.22	30.41	30.78	35.17	34.35	30.5	30.82	28.1	28.13	33.21	32.61	31.42	31.7	34	33.84	33.21	34.99	32.5	32.52	35.92	40	35.21	34.5	36.08	37.54	40	36.65
GLI1-Hs01110768_m1	27.77	27.27	26.72	26.47	40	35.95	28.39	27.91	31.93	33.1	29.26	29.09	29.62	29.76	29.31	29.33	29.67	29.4	29.31	30.07	28.57	28.08	28.19	28.4	29.46	29.13	25.65	25.75
GLI2-Hs01119974_m1	26.86	26.95	26.07	26.28	35.12	36.72	28.27	28.27	28.91	29.19	25.68	25.8	25.5	25.3	26.1	26.06	25.45	25.66	26.07	26.06	25.03	25.04	24.76	24.62	26.38	26.42	25.16	25.36
GLI3-Hs00609233_m1	27.79	27.65	26.27	26.24	26.63	26.63	25.96	25.98	27.29	27.3	24.2	24.07	23.9	23.98	24.38	24.27	24.23	24.6	24.97	25.06	24.66	24.6	23.36	23.51	24.46	24.74	24.42	24.6
GNL3-Hs00205071_m1	23.16	23.33	23.3	23.35	27.39	27.46	25.98	26.07	25	25.04	22.82	22.9	22.84	22.86	23.53	23.58	23.51	23.64	24.41	24.36	24.19	24.28	24.2	24.37	23.56	23.86	24.11	24.06
GSX1-Hs00793699_g1	40	40	40	34.73	40	40	31.79	31.5	40	35	40	40	31.94	31.6	35.07	36.12	27.19	27.04	30.18	30.98	28.5	28.81	29.62	29.53	40	40	40	40
GSX2-Hs00370195_m1	40	40	32.07	32.18	40	40	40	40	40	40	40	40	37.15	40	37.12	40	40	40	31.58	30.99	38.31	37.08	40	40	40	40	40	40
HES1-Hs00172878_m1	29.34	29.46	29.22	29.13	27.66	27.5	26.66	26.41	28.83	28.88	26.77	26.65	26.44	26.51	27.17	27.15	27.09	26.96	26.41	26.42	26.37	26.35	26.64	26.7	27.36	27.6	25.84	25.91
HES5-Hs01387463_g1	33.31	34.39	30.86	30.84	28.02	28.04	27.97	27.83	31.45	30.94	34.22	36.36	28.58	28.79	33.02	33.59	24.92	24.87	22.94	22.97	23.07	23.11	23.72	23.98	27.37	27.58	26.13	26.31
HEY1-Hs00232618_m1	32.95	32.71	31.78	31.95	30.14	29.94	27.66	27.69	27.95	27.86	30.76	30.86	31.15	31.36	32.56	32.22	33.28	33.78	27.87	27.91	27.62	27.65	29.98	30.05	31.44	31.66	30.66	30.7
HNF1B-Hs00172123_m1	30.5	30.48	33.98	34.86	40	40	40	40	40	40	40	40	40	40	40	40	40	40	40	40	40	40	40	40	40	40	40	40
HOXA2-Hs00534579_m1	40	37.34	38.62	40	40	40	34.33	33.75	40	40	28.02	27.68	27.72	27.7	30.71	30.28	40	40	25.57	25.49	26.13	25.97	40	40	34.87	34.65	39.16	40
HOXB2-Hs01911167_s1	29.99	29.95	27.6	27.44	26.38	26.24	29.36	29.08	26.83	26.77	23.93	23.85	21.77	21.98	25.03	25.16	36	40	22.76	22.83	22.97	23.09	40	36.41	24.46	24.58	27.31	27.4
HOXB4-Hs00256884_m1	28.46	28.77	30.12	30.28	27.16	26.96	28.71	28.67	31.35	31.03	31.22	30.48	32.56	32.49	40	40	40	40	22.91	22.91	24.29	24.33	40	40	34.7	34.17	40	40
HOXB8-Hs00256885_m1	40	40	40	40	40	40	40	36.61	40	40	32.53	32.81	40	40	40	40	40	40	35.51	34.74	35.41	35.23	40	40	40	40	40	40
HOXB9-Hs00256886_m1	27.51	27.62	30.66	30.66	40	40	35.96	40	40	40	29.64	29.54	40	35.98	40	40	40	40	33.01	33.32	30.59	30.76	40	40	34.22	40	40	40

Gene	Edi-2	Edi-2	DF-3F	DF-3F	CB541	CB541	CB660	CB660	CB192	CB192	AF22	AF22	AF23	AF23	AF24	AF24	PKb	PKb	PKc	PKc	Pka	Pka	PKd	PKd	I3	I3	H9.2	H9.2
HOXD3-Hs00232506_m1	40	40	35.98	40	40	40	32.59	32.25	40	40	34.19	35.42	40	40	40	40	40	40	26.83	26.74	33.47	32.97	40	40	40	40	32.19	32.68
HOPX-Hs00261238_m1	31.87	31.26	32.64	33.03	28.45	28.2	27.14	27.24	28.16	27.94	35.87	40	40	40	40	36.33	33.63	35.13	27.49	27.46	33.9	34.62	35.98	40	40	40	40	40
ID2-Hs00747379_m1	25.56	25.5	23.82	23.68	29.24	29.48	25.44	25.4	30.48	30.41	24.67	24.41	24.85	24.97	24.03	23.92	22.72	22.75	21.79	21.76	22.95	22.89	23.52	23.89	23.38	23.47	23.45	23.62
ID3-Hs00171409_m1	22.98	23.24	21.92	21.89	27.41	27.44	21.97	22.1	28.99	29.03	20.34	20.39	21.92	21.93	21.88	21.83	23.4	23.31	19.51	19.6	21.14	21.15	22.03	22.03	21.33	21.31	20.79	20.77
IRX2-Hs01383002_m1	24.19	24.04	24.18	24.06	25	25.12	25.54	25.54	29.19	29.07	22.16	22.2	21.71	21.48	22.88	22.73	22.26	21.95	25.85	25.66	25.09	25.01	21.98	21.73	23.06	23.11	22.15	22.11
IRX3-Hs00735523_m1	27.99	28.05	27.13	27.24	27.53	27.51	26.91	26.98	24.14	24.1	25.13	24.95	24.15	24.11	24.56	24.67	22.88	22.93	22.68	22.88	22.98	22.79	22.97	23.01	24.08	24.09	24.49	24.47
ISL1-Hs00158126_m1	32.67	32.14	30.33	30.28	40	40	34.21	34.03	40	40	32.83	32.42	35.43	35.7	37.03	36.02	35.69	33.3	34.59	34.97	33.1	33.11	40	40	40	40	34.67	36.39
LEF1-Hs01547248_m1	35.96	34.9	37.84	39.45	40	40	31.11	30.75	32.15	31.67	31.96	32.69	32.67	33.11	32.64	33.43	40	36.38	33.33	35.98	34.77	34.95	36.47	35.32	40	40	37.16	36.72
LGR5-Hs00173664_m1	29.79	29.79	34.57	34.59	40	40	28.55	28.31	33.74	33.49	30.4	30.41	32.3	32.16	31.56	31.68	29.96	29.95	27.17	27.17	28.17	28.2	27.18	27.41	28.6	28.77	29.09	29.21
LHX2-Hs00180351_m1	33.86	33.43	31.62	31.95	28.84	28.76	25.54	25.47	26.59	26.47	30.42	30.25	31.95	31.5	31.6	31.94	32.93	32.8	31.04	30.97	36.48	36.48	37.04	37.02	34.05	34.13	36.95	36.24
LHX4-Hs00369146_m1	29.65	29.93	30.02	29.78	36.52	40	36.84	40	35.72	35.28	40	40	40	40	40	40	34.94	34.45	27.06	26.77	29.35	29.39	34.76	35.94	36.6	36.47	34.7	34.14
LHX6-Hs00232660_m1	30.55	30.83	31.64	31.74	33.48	33.33	32.39	31.82	40	40	31.42	31.48	33.96	34.36	32.86	33.14	40	40	40	40	40	40	35.18	40	27.68	27.56	37.13	36.3
LHX8-Hs00418293_m1	32.6	32.92	32.62	32.72	33.95	40	28.58	28.5	40	40	29.8	29.71	40	40	30.28	30.18	28.9	29.53	31.55	31.34	40	40	40	40	40	40	40	40
LIX1-Hs00404153_m1	30.78	30.85	30.06	29.95	34.4	34.41	27	27.12	32.96	33	27.95	27.94	27.29	27.38	27.08	27.34	25.14	25.46	30.42	30.21	28.48	28.57	24.67	24.56	28.37	28.45	27.22	27.25
LMO3-Hs00375237_m1	35.46	34.37	32.87	33.4	29.04	29.04	26.37	26.51	33.6	33.58	33.28	32.76	30.97	31.21	30.54	30.96	27.14	27.78	25.47	25.46	26.68	26.71	28	28.15	33.24	32.82	26.75	26.82
LMX1A-Hs00602600_m1	40	35.62	36.25	36.19	40	40	40	40	40	40	40	40	40	40	40	40	40	40	40	40	40	40	40	40	40	40	40	40
LMX1B-Hs01059598_m1	40	40	40	40	40	40	40	40	40	40	40	40	40	40	40	40	40	40	40	40	40	40	40	39.21	40	40	40	40
MAPT-Hs00213487_m1	40	40	40	40	40	40	40	40	40	40	40	40	40	40	40	40	40	40	40	40	40	40	40	40	40	40	40	40
MBP-Hs00921943_m1	34.38	33.64	33.05	32.41	35.94	36.03	35.39	33.84	40	40	31.96	32	30.8	31.14	34.04	34.91	33.96	34.67	40	33.98	32.72	32.28	31.46	31.76	32.02	32.41	32.12	32.4
MDM2-Hs99999008_m1	24.67	24.82	25.57	25.48	26.56	26.43	26.23	26.33	25.66	25.84	24.36	24.33	24.09	24.29	24.81	24.78	25.18	25.74	25.4	25.43	24.88	25.04	24.04	24.44	26.7	26.7	24.36	24.66
MDM4-Hs00159092_m1	27.33	27.42	26.98	27.12	29.43	29.34	27.97	27.89	27.94	27.96	25.98	25.92	25.75	25.88	26.25	26.51	26.38	26.34	26.32	26.57	26.15	26.28	25.33	25.94	26.86	26.96	25.71	25.75
MEF2C-Hs00231149_m1	33.2	34.29	31.26	30.69	30.19	30.28	29.1	29.17	29.52	29.5	27.1	27.01	26.31	26.16	28.01	27.98	30.13	29.91	29.18	29.3	29.27	29.64	29.81	29.65	30.24	30.54	29.19	29.06
MEIS1-Hs00180020_m1	32.12	32.02	30.82	30.8	30.6	30.69	29.75	29.74	25.96	25.91	25.03	24.97	24.32	24.39	26.6	26.44	25.04	25.25	23.65	23.7	23.62	23.64	25.48	25.35	25.67	25.68	25.75	25.67
MEIS2-Hs00542638_m1	29.04	29.3	27.71	27.45	32.63	32.24	27.03	27.04	25.62	25.72	24.62	24.63	24.44	24.35	26.23	26.44	28.91	29.25	22.15	21.88	22.73	22.68	27.1	27.3	24.95	24.88	24.29	24.57
MELK-Hs00207681_m1	25.87	25.96	26	26.41	29.21	28.82	27.47	27.4	26.65	26.53	24.56	24.31	23.92	24.13	25.12	25.27	25.04	25.2	25.4	25.75	25.94	25.9	25.54	25.63	26.42	26.81	25.74	25.88
MMRN1-Hs00201182_m1	40	40	30.84	31.26	29.02	29.04	28.11	28.03	40	40	23.49	23.48	24.5	24.32	24.32	24.38	23.32	23.4	27.8	27.82	25.05	25	23.4	23.39	25	25.1	23.68	23.58
MSI1-Hs01045894_m1	24.95	25.1	24.72	24.88	24.95	25.1	23.62	23.74	23.93	24.01	22.32	22.52	21.98	22.15	22.64	22.71	22.22	22.23	22.54	22.69	22.4	22.43	21.17	20.99	22.8	22.84	21.55	21.59
MSX1-Hs00427183_m1	33.37	33.4	40	39.05	33.38	33.2	34.5	33.87	37.22	36.53	32.46	32.23	34.14	34.06	33.59	33.73	33.13	33.07	34.06	34.19	33.56	33.92	36.55	37.3	36.29	36.12	35.74	35.28
MXD3-Hs01557564_m1	27.83	27.65	25.65	25.51	28.22	27.9	26.27	26.16	26.25	26.13	24.54	24.6	24.51	24.36	24.56	24.68	23.3	23.49	23.94	23.99	24.5	24.45	24.19	24.38	24.76	24.66	24.18	24.18
MYC-Hs00905030_m1	24.85	24.94	25.46	25.46	28.54	28.44	27.84	28.02	25.05	25.04	24.44	24.29	25.48	25.87	25.74	25.74	27.52	27.78	27.22	27.36	27.55	27.79	24.27	24.37	28.39	28.67	26.47	26.65
MYCN-Hs00232074_m1	24.02	23.87	23.75	23.72	30.42	30.12	26.28	26.3	35.56	40	23.55	23.68	22.96	23.19	24.01	23.99	23.92	24.03	24.32	24.39	23.38	23.52	23.47	23.71	24.6	24.57	23.01	22.79
NEFM-Hs00193572_m1	27.86	27.83	26.86	26.67	36.64	40	23.97	24.02	31.79	31.07	20.51	20.62	24.81	24.89	23.28	23.25	25.98	25.87	22.63	22.88	21.28	21.36	24.5	24.63	21.5	21.53	22.68	22.52
NEUROG1-Hs00246211_s1	36.07	37.01	31.46	32.5	40	40	32.89	32.41	40	36.95	34.11	35.14	36.94	34.71	40	40	30.2	30.06	28.46	28.84	28.18	28.21	32.35	32.85	35.03	35.57	36.07	39.23
NEUROG2-Hs00702774_s1	35.12	33.4	31.85	31.46	40	35.55	29.66	29.86	35.12	40	35.17	40	31.42	31.67	35.15	35.08	28.73	28.4	28.78	28.69	30.4	31.35	27.95	28.04	33.66	33.9	29.76	30
NFE2L1-Hs00231457_m1	24.65	24.71	24.81	24.73	26.09	26.13	25.57	25.72	25.21	25.22	23.5	23.78	22.93	22.75	23.82	24.09	24.93	25.14	24.77	24.81	24.63	24.71	24.14	24.46	25.07	25.42	24.01	24.82
NF1-Hs00169714_m1	26.33	26.48	26.08	25.94	27.33	27.14	26.15	26.17	26.33	26.41	25.18	25.18	24.4	24.61	25.3	25.28	24.73	24.83	24.73	24.6	24.63	24.84	26.07	25.85	25.77	25.84	26.61	26.58
NFIA-Hs00325656_m1	29.81	29.56	27.12	26.99	32.24	32.25	25.51	25.64	29.36	29.27	28.55	28.78	28.43	28.46	29.12	29.4	29.95	29.45	24.55	24.67	26.96	27.11	36.64	35.44	34.38	33.5	35.14	34.82
NFIB-Hs01029176_m1	28.48	28.52	27.56	27.35	25.65	25.64	25.01	25.15	24.26	24.21	26.68	26.91	28	28.08	27.22	27.07	29.2	29.22	25.86	25.89	27.78	27.85	32.13	32.93	30.58	30.37	30.97	30.72
NFIX-Hs00231172_m1	26.41	26.54	27.14	27	24.63	24.62	24.13	24.03	22.65	22.65	29.25	29.41	29.59	29.35	29.33	29.89	30.07	29.78	27.83	27.71	32.04	31.98	32.23	32.33	34.68	33.75	35.08	33.89
NKX2-1-Hs00163037_m1	34.53	33.85	40	40	26.44	26.53	27.32	27.44	40	40	40	40	40	40	40	40	40	40	40	40	40	40	40	40	40	40	40	40

Gene	Edi-2	Edi-2	DF-3F	DF-3F	CB541	CB541	CB660	CB660	CB192	CB192	AF22	AF22	AF23	AF23	AF24	AF24	PKb	PKb	PKc	PKc	Pka	Pka	PKd	PKd	I3	I3	H9.2	H9.2
NKX2-2-Hs00159616_m1	33.17	33.19	37.65	35.65	32.99	33.11	33.15	32.43	34.98	34.8	40	40	40	40	40	40	40	40	35.23	37.16	33.65	33.93	40	40	40	36.24	25.66	26.01
NKX6-1-Hs00232355_m1	31.92	32.02	31.08	31.09	36.01	37.42	27.76	27.64	40	37.69	28.88	28.79	30.74	30.78	27.02	27.15	30.26	30.5	22.65	22.77	23.04	23.04	28.75	28.82	25.01	25.08	23.64	23.66
NR1H2-Hs00173195_m1	27.66	27.45	27.12	27.17	29.11	29.15	28.4	28.31	27.3	27.52	27.48	27.49	26.8	26.8	27.98	28.05	27.39	27.34	27.43	27.44	27.81	27.86	26.85	27.05	27.93	28.03	27.53	27.46
NR1H4-Hs00231968_m1	40	40	40	40	40	40	40	40	36.43	34.23	35.49	36.3	40	40	40	40	34.28	33.22	34.5	33.88	34.72	34.94	40	36.56	40	40	40	35.82
NR2E1-Hs00172664_m1	34.71	34.13	40	36.39	35.42	35.72	28.51	28.52	32.88	33.56	40	40	40	40	40	40	40	40	40	40	40	40	40	37.77	40	40	40	40
NR2F1-Hs00818842_m1	34.85	36.64	28.04	28.24	25.69	25.78	22.91	23.09	24.27	24.41	21.86	21.81	19.95	19.98	21.61	21.66	19.91	19.98	21.52	21.67	21.72	21.79	21.4	21.63	20.51	20.74	20.81	20.86
NR2F2-Hs00819630_m1	34.92	33.45	28.4	28.41	25.01	24.99	22.82	22.86	24.84	24.9	21.78	21.75	22.17	22.07	22.08	22.08	21	20.91	23.11	23.07	23.48	23.49	22.17	22.01	21.97	21.88	21.7	21.59
NR4A2-Hs00428691_m1	32.49	32.97	33.08	33.54	40	37.23	32.75	33.37	34.66	35.35	32.97	32.85	32.02	31.92	33.01	33.47	31.21	31.73	32.7	31.98	34	33.36	31.93	32.25	30.81	31.23	29.82	29.95
NR4A3-Hs00545009_g1	33.78	33.44	30.73	31.16	31.91	32.02	32.42	32.73	32.9	33.5	31.48	31.11	28.99	29.28	30.22	30.16	28.51	28.61	30.83	31.04	30.81	31.55	25.49	25.54	29.41	29.01	28.53	28.69
OLIG1-Hs00745341_s1	35.96	40	34.15	35.78	24.74	24.7	28.27	28.14	29.35	29.39	27.38	27.2	25.81	25.63	30.29	29.89	30.6	30.57	31.35	31.35	28.66	28.87	34.79	35.19	27.75	27.87	23.12	23.17
OLIG2-Hs00300164_s1	36.38	40	33.72	34.13	27.21	27.35	29.08	28.98	29.61	29.67	28.86	29.42	27.28	27.62	31.19	32.07	31.85	32.23	31.86	32.45	30.11	30.57	38.19	36.16	28.09	28.26	24.99	25
OTX1-Hs00951099_m1	30.48	30.91	30.09	30.29	33.54	34.64	30.01	29.77	25.65	25.78	29.36	29.44	33.89	32.91	29.26	29.7	24.9	25.11	32.54	32.67	28.74	28.78	27.36	27.53	26.26	26.13	25.15	25.07
OTX2-Hs00222238_m1	24.09	23.87	23.62	23.68	28.13	28.19	27.17	26.99	24.91	24.99	40	36.97	36.57	40	40	36.93	33.78	34.45	30.48	30.65	36.84	40	40	36.29	40	36.2	34.44	36.37
PAX2-Hs01057416_m1	35.11	36.75	40	34.72	40	40	28.66	28.5	40	40	35.52	40	40	40	40	40	40	40	28.87	28.99	35.36	34.37	37.87	40	40	40	40	40
PAX3-Hs00992437_m1	38.2	36.43	34.26	38.1	40	40	33.41	33.93	40	40	28.29	28.4	25.75	25.86	28.26	28.71	34.6	34.38	29.81	29.83	29	29.04	27.9	28.48	31.81	31.78	40	40
PAX6-Hs00240871_m1	29.69	29.76	31.15	30.41	34.33	36.34	27.54	27.55	27.73	27.84	27.24	27.5	24.23	24.27	25.75	25.57	22.23	22.19	22.7	22.85	23.23	23.32	24.23	24.37	24.26	24.27	24.62	24.76
PAX7-Hs00242962_m1	35.69	34.49	40	40	40	40	31.65	31.76	40	40	36.55	35.68	32.17	32.24	40	40	34.73	36.36	31.5	31.45	31.16	31.52	27.81	27.87	40	40	40	40
PDGFRA-Hs00183486_m1	29.81	29.97	33.77	32.35	40	40	33.48	32.49	31.98	33.8	28.98	28.97	27.98	28.05	28.19	28.26	29.56	29.54	28.85	28.91	28.87	28.83	40	40	40	40	40	40
PDGFRB-Hs00387364_m1	28.46	28.56	28.76	29.05	27.5	27.51	24.73	24.79	28.79	28.93	24.48	24.87	24.99	25.02	25.39	25.46	26.04	26.39	24.38	24.65	25.42	25.75	26.28	26.43	25.15	25.32	24.62	24.74
PELP1-Hs00413506_gH	23.69	23.6	23.49	23.37	26.79	26.89	25.71	25.71	25.62	25.62	23.61	23.64	22.55	22.58	24.33	24.34	23.23	23.32	23.75	23.76	24.02	24.12	23.24	23.52	24.71	24.7	23.55	23.41
PHOX2A-Hs00605931_mH	32.53	32.39	30.58	31.23	40	40	40	36.21	36.98	34.43	32.66	33.66	28.61	28.92	31.8	32.15	32.53	32.96	31.04	31.57	29.73	29.79	33.88	34.42	30.26	30.31	30.52	30.91
PITX3-Hs00374504_m1	32.62	31.69	31.19	30.9	40	40	31.27	31.48	40	40	32.68	40	33.42	33.65	32.72	33.7	30.93	30.79	33.88	35.56	32.44	32.92	34.49	35.79	33.06	32.51	34.79	34.37
PKNOX1-Hs00231814_m1	26.75	26.91	26.69	26.61	29.27	29.67	28.16	28.32	27.54	27.66	26.15	26.19	26.52	26.46	27.24	27.43	27.54	27.53	26.17	26.06	26.27	26.27	26.5	26.55	27.98	27.94	26.52	26.75
PLAG1-Hs00231236_m1	32.57	33.65	30.13	30.03	33.71	32.6	30.99	31.65	33.52	34.06	29.23	29.56	29.65	30.09	29.69	29.82	29.74	29.49	30.52	29.95	29.18	29.06	30.55	30.81	29.73	29.86	29.25	29.06
PMP2-Hs00160204_m1	40	40	40	37.21	35.98	35.98	32.97	33.13	30.03	29.95	40	35.59	40	40	40	35.65	36.64	37.33	31.76	31.96	30.08	30.23	36.32	35.46	40	40	35.26	36.55
POU3F1-Hs00538614_s1	32.48	32.31	31.4	31.51	32.76	32.64	32.66	33.01	36.3	36.72	27.05	27.17	26.53	26.56	28.4	28.46	28.68	28.82	31.86	31.94	31.23	31.11	27.46	27.41	28.42	28.18	27.55	27.36
POU3F2-Hs00271595_s1	34.95	34.68	27.85	28.01	27.52	27.77	27.5	27.6	24.56	24.65	23.4	23.48	22.23	22.27	23.74	23.81	22.58	22.62	22.95	23.07	22.98	23.02	22.89	23.05	23.75	24.02	23.54	23.63
POU3F3-Hs00275987_s1	36.13	38.63	32.45	32.76	27.52	27.35	28.51	28.59	27.51	27.48	25.82	25.78	25.62	25.56	26.21	26.07	26.66	26.56	26.72	26.71	25.92	25.7	25.29	25.07	27.5	27.33	25.31	25.28
POU3F4-Hs00264887_s1	35.77	40	30.91	30.92	40	40	27.97	27.83	29.56	29.56	26.61	26.59	27.1	26.97	29.39	29.49	24.33	24.25	25.69	25.57	40	40	24.93	24.82	25.68	25.7	29.77	29.44
POU4F1-Hs00366711_m1	35.89	40	33.63	37.97	40	40	36.26	36.28	40	36.29	35.07	36.51	35.29	34.4	36.35	35.53	34.99	35.56	35.04	33.28	34.17	33.27	34.49	34.03	36.16	34.72	34.68	34.22
POU6F1-Hs00231276_m1	29.43	29.49	29.61	29.51	33.17	32.73	30.87	30.75	30.51	30.42	29.73	29.57	29.57	29.7	30.34	30.36	29.29	29.49	28.13	28.36	28.87	28.95	29.5	30.26	29.7	29.93	29.64	29.98
PPP1R1B-Hs00259967_m1	30.7	30.58	28.09	28.06	35.75	38.03	33.1	32.84	34.58	34.6	37.94	38.08	33.23	33.7	36.3	34.84	35.84	33.99	31.94	31.93	36.75	37.97	38.42	38.47	35.53	35.66	38.29	37.93
PROM1-Hs01009259_m1	26.61	26.77	27.31	26.83	40	40	31.16	30.99	29.88	29.6	25.39	25.29	25.75	25.82	26.1	26.17	26.01	26.11	25.13	24.92	26.61	26.59	24.27	24.5	26.06	26.69	24.71	25.32
PTEN-Hs02621230_s1	28.53	28.75	28.01	28.32	27.28	27.38	27.97	27.94	28.31	28.28	27.08	27.05	26.84	26.88	27.82	27.84	27.29	27.49	27.35	27.46	27.51	27.37	27.79	27.98	27.25	27.66	28.36	28.39
PTN-Hs01085691_m1	24.08	24.02	21.66	21.72	22.98	23.04	21.64	21.63	19.34	19.37	16.59	16.87	17.39	17.54	17.6	17.85	19.09	18.97	20.15	20.36	21.01	21.02	19.05	19.01	20.52	20.47	20.61	20.75
PVALB-Hs00161045_m1	32.82	33.1	29.86	30.09	40	40	35.16	35.25	40	40	26.49	26.46	25.75	25.43	26.08	25.96	27.11	26.8	40	35.51	36.08	36.25	29.22	29.18	27.12	26.81	26.36	26.56
RB1-Hs00153108_m1	26.23	26.27	25.56	25.64	25.88	25.97	25.53	25.57	24.99	25.15	24.38	24.52	23.87	23.87	24.98	24.85	24.06	23.95	23.88	23.79	24.02	23.93	24.85	24.98	24.97	25.22	25.18	25.07
REST-Hs00958503_m1	23.15	23.01	23.83	23.8	26.19	26.09	25.25	25.4	25.16	25.04	23.57	23.53	23.67	23.55	24.41	24.25	24.04	23.97	24.96	24.83	25.26	25.17	24.96	24.98	25.26	24.98	25.71	25.67
RFX3-Hs00231292_m1	27.13	27.08	26.71	26.84	26.83	26.84	25.87	25.85	26	25.94	25.07	24.96	24.26	24.28	25.08	25.21	23.91	24.07	24.4	24.59	24.35	24.44	23.78	23.81	25.47	25.4	24.48	24.4
RSPO3-Hs00262176_m1	29.4	28.99	33.66	34.02	36.58	40	36.99	35.16	40	40	31	30.94	33.66	32.99	34.25	34.25	34.9	35.1	32.82	32.25	33.09	34.45	33.97	32.96	36.74	34.85	36.78	40

Gene	Edi-2	Edi-2	DF-3F	DF-3F	CB541	CB541	CB660	CB660	CB192	CB192	AF22	AF22	AF23	AF23	AF24	AF24	PKb	PKb	PKc	PKc	Pka	Pka	PKd	PKd	I3	I3	H9.2	H9.2
RUNX3-Hs00231709_m1	30.73	30.95	30.87	31.4	40	40	40	40	40	40	36.9	36.27	35.41	36.04	36.92	34.89	34.82	34.02	36.7	36.01	33.68	33.36	40	40	35.84	35.7	37.69	36.92
RXRA-Hs00172565_m1	25.61	25.67	25.42	25.68	26.89	26.93	26.09	26.07	25.94	26	24.32	24.24	24.27	24.28	24.9	24.93	24.89	24.89	24.75	24.73	24.86	24.83	24.23	24.27	25.42	25.46	24.37	24.4
RXRG-Hs00199455_m1	30.34	30.58	32.84	33.93	40	40	34.57	35.24	40	40	35.88	36	33.55	35.19	34.35	35.11	32.6	33.47	29.17	29.18	30.61	30.43	40	40	40	40	40	40
S100B-Hs00902901_m1	35.73	34.95	29.18	29.1	22.72	22.98	25.25	25.27	24.55	24.71	26.11	26.22	22.95	23.14	26.44	26.85	27.26	27.5	25.38	25.61	25.84	25.8	28.56	28.96	27.63	27.62	29.76	30.03
SHC1-Hs00427539_m1	23.27	23.32	23.64	23.69	24.84	24.87	23.99	23.94	23.05	23.3	21.76	21.59	22.33	22.37	23.23	22.98	24.8	24.55	25.53	25.68	24.57	24.82	24.63	24.27	24.59	24.87	23.08	23.31
SHC3-Hs00179650_m1	27.66	27.24	28.47	28.35	26.87	26.95	25.96	25.96	26.92	26.83	24.25	24.39	24.55	24.36	24.83	24.8	24.61	24.44	24.86	24.69	24.66	24.49	25.32	25.41	24.92	24.89	24.66	24.51
SHH-Hs01123832_m1	33.07	33.13	35.42	37.2	32.81	32.2	28.76	28.69	35.66	35.86	40	40	40	40	40	40	40	40	40	40	40	40	40	40	40	40	40	40
SIX3-Hs00193667_m1	35.87	34.48	30.8	30.71	24.77	24.69	22.92	22.79	32.5	35.52	40	40	36.31	40	36.4	34.86	27.17	27.05	30.52	31.33	40	40	34.91	35.91	33.36	32.99	33.69	34.11
SLC1A1-Hs00188172_m1	28.32	28.25	29.06	28.8	40	34.51	31.84	31.87	30.8	30.46	28.24	28.19	31.29	30.83	29.17	28.95	32.33	32.67	32.66	32.7	33.54	34.56	35.75	35.16	31.42	31.49	29.15	29.1
SOX10-Hs00199510_m1	32.82	32.6	31.31	31.11	38.15	40	40	38.07	40	40	39.77	40	38.2	40	40	40	40	40	39.42	39.54	39.09	39.44	40	40	38.93	38.13	28.68	40
SOX1-Hs01057642_s1	40	40	36.32	35.05	31.54	31.56	31.1	31.04	32.95	33.03	30.94	31.08	30.82	30.89	29.84	29.74	27.98	27.94	29.51	29.48	30.27	30.71	28.17	28.82	29.43	29.18	29.26	29.7
SOX21-Hs00359892_s1	34.16	34.18	31.64	31.99	32.42	32.76	33.19	32.85	31.67	31.84	33.64	34.03	30.59	30.81	32.44	32.65	29.19	29.52	31.52	31.5	31.66	31.86	29.98	30.41	31.23	31.13	31.31	31.48
SOX2-Hs00602736_s1	27.79	28.3	27.51	27.1	29.27	29.16	28.07	27.97	28.38	28.16	27.14	26.55	26.6	26.37	26.96	27.13	26.21	26.38	26.14	26.94	27.24	27.29	26.24	26.78	27.53	27.8	26.91	27.62
SOX3-Hs00271627_s1	33.6	32.7	33.81	33.46	36.73	36.17	35.07	33.98	40	40	35.05	34.87	29.7	29.67	33.17	32.34	28.35	27.64	27.7	27.04	27.49	26.94	26.03	25.55	28.62	27.9	27.9	27.3
SOX5-Hs00753050_s1	31.11	30.96	29.13	29.36	27.19	27.13	26.1	26.07	28.5	28.44	26.2	26.19	26.19	26.4	26.76	26.67	26.17	26	26.85	26.94	26.78	26.92	25.77	26.08	26.92	27.01	26.5	26.56
SOX6-Hs00264525_m1	32.41	32.67	34.23	33.77	31.28	31.98	28.52	28.55	30.64	30.69	27.62	27.54	27.99	27.91	28.78	28.58	28.98	29.32	27.48	27.44	30.63	30.7	28.82	28.86	30.23	30.6	28.97	28.95
SOX8-Hs00232723_m1	30.96	30.68	27.52	27.73	28.21	28.18	26.68	26.74	27.28	27.15	26.14	26.08	26.61	26.75	27.4	27.36	27.38	27.48	25.86	25.95	25.81	25.97	25.08	25.63	26.19	26.49	23.98	24.15
SOX9-Hs00165814_m1	28.88	28.98	25.85	25.82	24.96	25	24.22	24.23	24.15	24.15	21.92	21.98	22.43	22.43	23.66	23.51	23.99	23.97	22.43	22.29	23.48	23.49	25.71	25.73	24.69	24.72	24.85	24.7
SPARCL1-Hs00190740_m1	33.14	34.28	29.79	30.05	23.69	23.62	20.95	20.89	25.99	26.06	27.48	27.66	23.79	23.78	24.72	24.85	24	23.95	24.69	24.7	25.02	25.07	26.7	27.42	26.85	27	25.14	25
SREBF1-Hs01088691_m1	27.97	27.91	27.35	27.46	27.38	27.38	26.77	26.63	26.48	26.36	25.73	25.79	24.55	24.94	26.21	26.19	24.36	24.81	24.99	24.74	24.4	24.24	24.74	24.56	25.59	25.7	24.78	24.63
SYN1-Hs00199577_m1	29.24	29.34	28.44	28.6	30.3	30.13	30.3	30.39	31.94	32.58	27.77	27.98	27.09	27.32	29.13	29.37	29.29	29.51	26.81	26.79	27.01	27.22	30.17	30.12	29.12	28.75	29.52	29.81
SYP-Hs00300531_m1	32.95	33.27	30.24	30.32	32.86	32.74	31.03	31.03	33.47	34.25	31.38	31.99	30.36	30.75	31.05	31.67	30.25	30.14	27.98	28.1	27.69	27.63	28.29	28.53	30.33	30.5	31.49	31.28
TBR1-Hs00232429_m1	40	35.63	35.48	40	40	35.12	35.72	40	33.98	34.77	35.65	34.5	34.99	34.73	35.92	35.78	34.6	40	35.31	40	40	35.7	32.85	33.42	36.6	34.77	35.46	34.62
TCF12-Hs00175295_m1	25.58	25.36	24.8	25.12	26.1	25.97	25.38	25.34	25.05	25.12	23.47	23.39	23.28	23.33	23.89	23.98	23.68	23.79	23.74	23.76	23.97	23.96	23.53	23.52	24.73	24.74	23.95	23.98
TCF4-Hs00162613_m1	25.71	25.87	25.14	25.18	27.42	27.49	26.04	26.09	26.64	26.73	23.84	23.94	24.18	24.14	24.55	24.6	24.27	24.46	24.37	24.37	24.54	24.35	24.44	24.73	25.52	25.58	25.04	25.23
TCF7L2-Hs00181036_m1	24.99	24.97	23.31	23.45	25.3	25.26	24.63	24.58	24.89	24.76	23.23	23.21	22.81	22.98	23.6	23.79	22.71	22.75	23.48	23.52	23.81	23.9	23.29	23.42	23.68	23.78	23.63	23.56
TERT-Hs00972646_m1	29.69	29.73	27.16	27.28	40	40	40	40	40	40	32.51	32.41	35.48	34.41	30.73	30.97	29.42	29.68	34.12	33.32	31.85	31.99	30.87	30.35	30.22	30.51	29.22	29.25
TFDP1-Hs00955491_gH	25.02	24.89	25.76	24.95	27.25	27.47	26.12	26.31	25.35	25.31	23.76	23.62	23.61	23.14	24.6	24.06	24.44	24	24.82	24.73	24.97	24.99	23.75	23.41	25.39	25.29	24.7	24.4
TH-Hs00165941_m1	40	36.63	33.04	33.09	31.7	31.83	32.09	31.98	31.14	30.91	34.79	34.78	33.45	32.97	35.64	35.9	36.47	40	29.41	29.91	29.51	29.49	40	40	35.35	34.89	39.05	40
TP53-Hs00153340_m1	23.05	22.99	22.93	22.9	25.9	25.97	24.61	24.64	24.09	24.1	22	22.11	21.55	21.68	22.54	22.57	22.53	22.65	23.06	23.14	23.19	23.14	22.03	22.25	25.73	25.53	21.69	21.85
TSC22D4-Hs00229526_m1	25.37	25.41	24.99	25	26.74	26.72	25.9	25.84	25.8	25.77	24.46	24.56	23.82	23.86	24.92	25.43	24.47	24.67	24.7	24.91	25.07	24.99	25.33	25.39	25.11	25.25	25.56	25.53
TUBB3-Hs00964965_m1	40	40	40	34.95	36.66	40	35.94	36.97	35.53	40	40	36.97	36.95	40	37.05	36.78	36.55	37.15	34.42	36.39	34.67	35.03	34.81	35.15	40	40	35.29	36.96
ZBTB16-Hs00232313_m1	36.31	34.33	32.76	32.64	27.77	27.66	29.98	29.52	30.36	30.27	25.23	25.05	24.11	24.02	25.1	25.01	23.93	23.81	25.87	25.94	25.11	24.87	24.05	24.19	25.17	25.04	24.75	24.76
ZFHX3-Hs00199344_m1	30.15	29.6	28.84	28.88	30.22	30.79	27.81	27.78	27.68	27.91	25.87	25.97	25.18	25.16	25.13	24.96	24.22	24.22	24.48	24.61	24.63	24.49	24.38	24.16	25.44	25.34	25.35	25.4
